# Decreased proliferation of HepG2 liver cancer cells in vitro and exhibited proteomic changes in vivo in subjects with metabolic syndrome and metabolic dysfunction-associated steatotic liver disease who performed four-week dawn-to-dusk dry fasting

**DOI:** 10.1186/s12014-025-09547-3

**Published:** 2025-06-24

**Authors:** Ayse L. Mindikoglu, Kristin Eckel-Mahan, Antone R. Opekun, Mustafa M. Alzubaidi, Zoe R. Crochet, Prasun K. Jalal, Sung Yun Jung

**Affiliations:** 1 Margaret M. and Albert B. Alkek Department of Medicine, Section of Gastroenterology and Hepatology, Baylor College of Medicine, Houston, TX USA; 2 Institute of Molecular Medicine, McGovern Medical School, University of Texas Health Science Center, Houston, TX USA; 3 Department of Integrative Biology and Pharmacology, McGovern Medical School, University of Texas Health Science Center, Houston, TX USA; 4 Department of Pediatrics, Division of Gastroenterology, Hepatology and Nutrition, Baylor College of Medicine, Houston, TX USA; 5 Department of Biochemistry and Molecular Pharmacology, Baylor College of Medicine, Houston, TX USA

**Keywords:** Dry fasting, Intermittent fasting, Dawn-to-dusk dry fasting, Diurnal fasting, Daytime fasting, Human serum, Liver cancer, Hepatoblastoma, Human serum proteome

## Abstract

**Background:**

Four-week dawn-to-dusk dry fasting (DDDF) was previously shown to have a potent anti-inflammatory effect and induce an anti-tumorigenic proteome in the serum and peripheral blood mononuclear cells in subjects without cancer. The study goal was to determine if serum obtained from these subjects without cancer who underwent 4-week DDDF has an anti-tumorigenic effect.

**Methods:**

HepG2 cells were treated with serum collected from four individuals with metabolic syndrome and metabolic dysfunction-associated steatotic liver disease (MASLD) and four healthy individuals who performed 4-week DDDF. The objective was to assess cell proliferation/viability in HepG2 cells treated with non-fasted and dry-fasted serum and determine proteomic changes in human serum. We comparatively performed 3-[4,5-dimethylthiazol-2-yl]-2,5-diphenyltetrazolium bromide (MTT) cell proliferation assay and untargeted proteomic analysis using nano ultra-high performance liquid chromatography coupled with tandem mass spectrometry.

**Results:**

Serum collected from 3 out of 4 subjects with metabolic syndrome and MASLD at the end of 4-week DDDF (dry-fasted serum/V2) significantly reduced proliferation/viability in HepG2 cells compared with the serum collected before 4-week DDDF (non-fasted serum/V1). A similar reduction effect on cell proliferation was not observed when HepG2 cells were treated with dry-fasted serum collected from healthy subjects. In addition to the in vitro changes observed, the following circulating gene protein products (GP) demonstrated significant increases or decreases in subjects with metabolic syndrome and MASLD after a 4-week DDDF regimen, compared with their GP levels before the 4-week DDDF: CD248 molecule (mean log2 fold = 8.124, *P* = 0.001), dipeptidyl peptidase 4 (mean log2 fold = 0.937, *P* = 0.027), lymphatic vessel endothelial hyaluronan receptor 1 (mean log2 fold = 1.054, *P* = 0.029), LDL receptor related protein 1 (mean log2 fold = 1.401, *P* = 0.031), and beta-2-microglobulin (mean log2 fold= -0.977, *P* = 0.033) at the end of 4-week DDDF compared with the GP levels before 4-week DDDF.

**Conclusion:**

This study demonstrated that dry-fasted serum collected from subjects with metabolic syndrome and MASLD decreased HepG2 cell proliferation in vitro and showed that proteomic changes occurred in vivo. These findings suggest that DDDF may be an effective intervention for inducing proteomic responses that could assist in the prevention and adjunct treatment of cancers associated with metabolic syndrome.

**Supplementary Information:**

The online version contains supplementary material available at 10.1186/s12014-025-09547-3.

## Introduction

A global epidemic of metabolic syndrome and metabolic dysfunction-associated steatotic liver disease (MASLD) is tightly related to obesity [1, 2]. Metabolic syndrome, a significant risk factor for developing systemic inflammation, endothelial dysfunction, and MASLD, is diagnosed when three or more of the five adverse clinical features are present, including increased waist circumference, insulin resistance, elevated blood pressure, high blood triglyceride, and low high-density lipoprotein levels [3–6]. Importantly, subjects with metabolic syndrome and MASLD are at significant risk for developing malignancies, including hepatocellular carcinoma and several other cancers (e.g., pancreatic and colon cancer) [7, 8]. Therefore, to confer anti-tumorigenic effects, an ideal therapeutic regimen for subjects with metabolic syndrome and MASLD should improve metabolic parameters, induce a robust anti-inflammatory effect, and correct intracellular molecular aberrancies.

Intermittent fasting has been shown to have an anti-inflammatory and anti-tumorigenic effect [9–13]. In the murine osteosarcoma model, food deprivation during the activity period was shown to have the most robust anti-tumor effect compared with food deprivation during the inactivity period and ad libitum feeding [13]. The results were linked to improved host control over the tumor, alterations in the tumor’s circadian rhythms, or a combination of both [13]. Water fasting cycles of 48 to 60 h delay tumor progression and increase sensitivity to chemotherapy drugs in murine melanoma, neuroblastoma, and breast cancer models [9]. This is consistent with the Warburg effect that represents metabolic stress and the reprogramming of protein translation that could be fatal to malignant cells [14–16]. In humans, a similar anticancer effect may be achieved through a shorter fasting cycle if fasting is done dry (without eating or drinking) from dawn to dusk over several consecutive days [10–12]. 

Our previous studies on non-cancerous individuals suggest that dry fasting from dawn to dusk can promote an anti-inflammatory and anti-tumor proteome response, increasing tumor-suppressing proteins and decreasing those linked to tumor promotion [10–12]. Therefore, to validate the anti-tumorigenic effect of dawn-to-dusk dry fasting (DDDF) observed in subjects without cancer, we hypothesized that serum collected at the end of 4-week DDDF (i.e., proteome-conditioned serum by 4-week DDDF/dry-fasted serum) could reduce proliferation/viability of HepG2 liver cancer cells compared with serum collected before 4-week DDDF (non-fasted serum) (in vitro experiment) and induce proteomic changes in the same subjects’ serum at the end of 4-week DDDF compared with serum collected before 4-week DDDF (in vivo experiment).

Several features of DDDF make it a superior alternative to prolonged water/wet fasting regimens (water and zero-calorie drinks are allowed during fasting) to achieve anticancer effect: DDDF is compliant with zeitgeber modulated (external cues) circadian rhythm because mealtimes occur precisely at dawn and dusk immediately before and after fasting window. Brief pulses of light at dawn and dusk are sufficient to entrain the central clock in the suprachiasmatic nucleus of the anterior hypothalamus and establish a 24-hour circadian rhythm, while mealtimes strongly entrain peripheral clocks [17, 18]. It is thought that peripheral clocks are more vulnerable to modulation by food-related stimuli, such that a relative desynchronization between the central and peripheral clocks might increase the risk of metabolic dysfunction [19]. Therefore, regularly taking meals at dawn and dusk would also align the peripheral circadian clock phase with the central clock phase entrained to local dawn and dusk, potentially mitigating the risks of metabolic syndrome [11, 12]. The DDDF model stands in contrast to skipping breakfast routine that has been employed in several time-restricted eating (TRE) regimens and having late-night eating that disrupts the circadian regulation of metabolism [20–25]. The energy intake is imbalanced by irregular cues that misalign the peripheral clocks with the central clock, normally entrained to local dawn and dusk cues [19, 20–25].

Dry fasting is reported to protect against skeletal muscle and vital body weight loss that can occur with water/wet fasting due to the degradation of proteins as alternative energy sources when glycogen stores are depleted during fasting [26]. In fact, a randomized clinical trial of a TRE regimen that allowed study subjects to have water, black coffee and tea during the fasting window led to substantial lean mass loss [25]. Water/wet fasting should not be considered an actual fasting regimen but a water/zero calorie drink-based diet [26, 27]. Drinking during fasting likely inhibits vasopressin secretion, independent of plasma osmolality status, which also appears sufficient to indirectly misalign peripheral clocks [28]. Vasopressin is a powerful stimulant of lipolysis and beta-oxidation of fatty acids [29]. The inhibition of vasopressin leads to the inhibition of adrenocorticotropic hormone, resulting in the subsequent suppression of epinephrine release [30, 31]. Epinephrine depletion has been shown to increase protein degradation during fasting, likely due to the inactivation of lipoprotein lipase; in turn, the fatty acids cannot be utilized as an energy source alternative to glucose during fasting [29, 32, 33]. Taken altogether, in theory, the act of drinking (water or any liquid) during water/wet fasting will shift energy production from glucose toward utilizing amino acids from skeletal muscle instead of fatty acids as a fuel substrate and result in muscle mass and vital body weight loss [26, 28]. 

DDDF is straightforward to comply with as the fasting window is from dawn to dusk if it is approached as part of a ritual [34], and this stands in contrast to 48 h to 60 h of continuous starvation.

With adequate hydration during the non-fasting window, DDDF was shown to have an excellent safety profile in various populations, including healthy individuals, subjects with increased body mass index, metabolic syndrome and MASLD, and renal transplant patients [10–12, 35–39]. Dry fasting is anticipated to induce metabolic water production (metabolism of 100 g of fat produces over 100 g of endogenous water) [26, 40–42]. An observational study involving 34 participants practicing daytime dry fasting found that both plasma and 24-hour urine osmolality were within the normal physiological range during the fasting period [38]. Dehydration during DDDF can be readily prevented with adequate hydration during non-fasting window, nutrition education, and avoiding conditions that can lead to dehydration during both non-fasting and fasting windows.

The practice of shorter fasting windows in the DDDF, particularly during the winter months, can minimize the likelihood of overeating during non-fasting periods and reduce the risk of refeeding syndrome. This potentially serious condition that can arise following extended periods of fasting [43]. 

To determine the anti-tumorigenic effect of 4-week DDDF, we treated HepG2 cells with serum collected from subjects with metabolic syndrome and MASLD and healthy subjects (control group) who performed 4-week DDDF. HepG2 cell line was initially reported to be isolated from a 15-year-old male with hepatocellular carcinoma [44, 45]. However, a later publication reported that the HepG2 cell line was derived from an epithelial hepatoblastoma, not a hepatocellular carcinoma [46]. Our primary aim was to determine whether serum collected at the end of 4-week DDDF (i.e., serum conditioned by 4-week DDDF/dry-fasted serum) can reduce proliferation/viability of HepG2 cells compared with serum collected before 4-week DDDF (non-fasted serum) (in vitro experiment). Our secondary aim was to determine proteomic changes in the same subjects’ serum at the end of 4-week DDDF compared with serum collected before 4-week DDDF (in vivo experiment).

## Methods

### Human serum collection

Prior to conducting this study, approval from the Institutional Review Board for Human Subject Research for Baylor College of Medicine and Affiliated Hospitals (Protocol number H-31612) was obtained. The inclusion and exclusion criteria, study design, and protocol H-31612 procedures to collect data and specimens from subjects with metabolic syndrome and MASLD, and healthy subjects who performed 4-week DDDF were previously reported [10, 11].

**Fig. 1:**
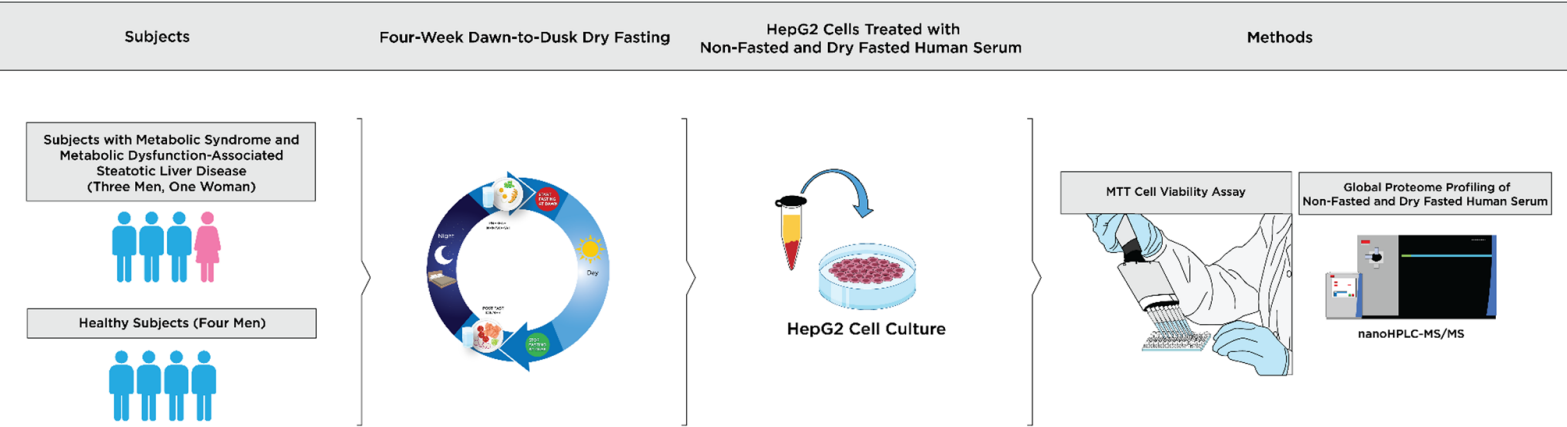
Subjects with metabolic syndrome and metabolic dysfunction-associated steatotic liver disease (MASLD) and healthy subjects fasted (strict dry fasting without food or drink intake) from dawn to dusk for over 14 h daily for 4 weeks. The serum was collected before 4-week DDDF (non-fasted serum) and at the end of 4-week DDDF (dry-fasted serum). After HepG2 cells were treated with non-fasted and dry-fasted serum, cell proliferation/viability in HepG2 cells was assessed by 3-[4,5-dimethylthiazol-2-yl]−2,5 diphenyltetrazolium bromide (MTT) cell proliferation/viability assay. An untargeted proteomic analysis was performed using nano ultra-high performance liquid chromatography-tandem mass spectrometry to determine the changes in proteins in non-fasted and dry-fasted serum collected from the same subjects

For the treatment of HepG2 cells, we used stored serum specimens from four subjects (three men and one woman, mean age 67 years [SD=6]) with metabolic syndrome and MASLD (Fig. 1) and four healthy male subjects (mean age=32 years [SD=6]) who practiced 4-week DDDF (Fig. 1). For both groups, we used the serum collected before 4-week DDDF (non-fasted serum) and at the end of 4-week DDDF (dry-fasted serum).

### Determination of HepG2 cell proliferation/viability: 3-(4,5-dimethylthiazol-2-yl)−2,5-diphenyltetrazolium bromide (MTT) assay

HepG2 cells were plated into flat-bottomed 96-well plates at a density of 5000 cells per well. Cells were incubated in a full-growth medium at 37 °C for 6 h until they were adherent to the plate. Media containing 20% V/V fetal bovine serum (FBS) (control) or human serum samples were added to each well. After 24 h, 15 µl of Dye Solution (CellTiter 96^®^ Non-Radioactive Cell Proliferation Assay #G4000, Promega, Madison, WI, USA) was added to each well, and the plates were incubated at 37 °C in a humidified 5% CO2 incubator for 4 h. 100 µl of Solubilization Solution/Stop mix was added to each well, and the plates were incubated in a CO2 incubator at 37 °C overnight. The plates were shaken on a horizontal shaker for 30 s to allow for complete dissolution, and then the absorbance of the plates was recorded at 570 nm wavelength using a 96-well plate reader. All the experiments were performed in triplicate.

### Human serum proteomic analysis

The proteomic profiling of serum using a mass spectrometer was conducted following established protocols [11]. Briefly, the serum samples were thawed at 37 °C, and 10 µl were collected for analysis. The 14 most abundant serum proteins were depleted using a commercial kit (Thermo Scientific, Cat# A36370). The remaining proteins were digested with trypsin on an S-Trap column (ProtiFi, NY), and the resulting peptides were vacuum-dried. These peptides were then fractionated into two pools using the high pH STAGE method [47] and analyzed with Thermo Scientific EASY-nLC 1000 coupled Orbitrap Fusion™ Tribrid™ Mass Spectrometer (Thermo Fisher Scientific). Peptide data were converted to quantifiable gene protein products (GPs) using the label-free, intensity-based absolute quantification (iBAQ) method and normalized to the final quantitative value (iFOT, defined as iBAQ of the individual identified protein divided by the total iBAQ of all identified proteins within one experiment) using proprietary software, as previously detailed [48]. 

### Statistical analysis

For statistical analyses, Microsoft^®^ Excel^®^ for Microsoft 365 MSO (Version 2504 Build 16.0.18730.20186) 64-bit software program (Microsoft, Redmond, WA, USA) and SAS software, Version 9.4 TS Level 1M7 X64_10PRO platform (SAS Institute Inc., Cary, NC, USA) [49] were used.

To determine statistically significant differences in HepG2 cell proliferation/viability at the end of 4-week DDDF compared with the HepG2 cell proliferation/viability before 4-week DDDF, independent two-tailed student’s t-tests (P value < 0.05) were performed.

The statistical analysis of proteomics was performed on three subjects with metabolic syndrome and MASLD, and three healthy subjects after removing one outlier subject from both groups. For analysis, log-converted intensity-based fraction of total iFOT values were used [10–12]. The GP level at the end of 4-week DDDF compared with the GP level before 4-week DDDF was considered differentially expressed if the GP level showed greater than or equal to 1.5-fold (log2 fold greater than or equal to 0.585) mean paired change with a P-value of < 0.05 [10–12].

We also used Omics Playground [50] for Prize-Collecting Steiner Forest (PCSF) algorithm to identify high-confidence subnetworks of highly correlated and differentially expressed proteins [51], with the goal of uncovering potential “driver” proteins that function as network hubs. The analysis was performed using the STRING protein-protein interaction network as a template.

## Results

### MTT cell proliferation/viability assays

#### MTT assay in HepG2 cells treated with non-fasted and dry-fasted serum collected from subjects with metabolic syndrome and MASLD

**Fig. 2:**
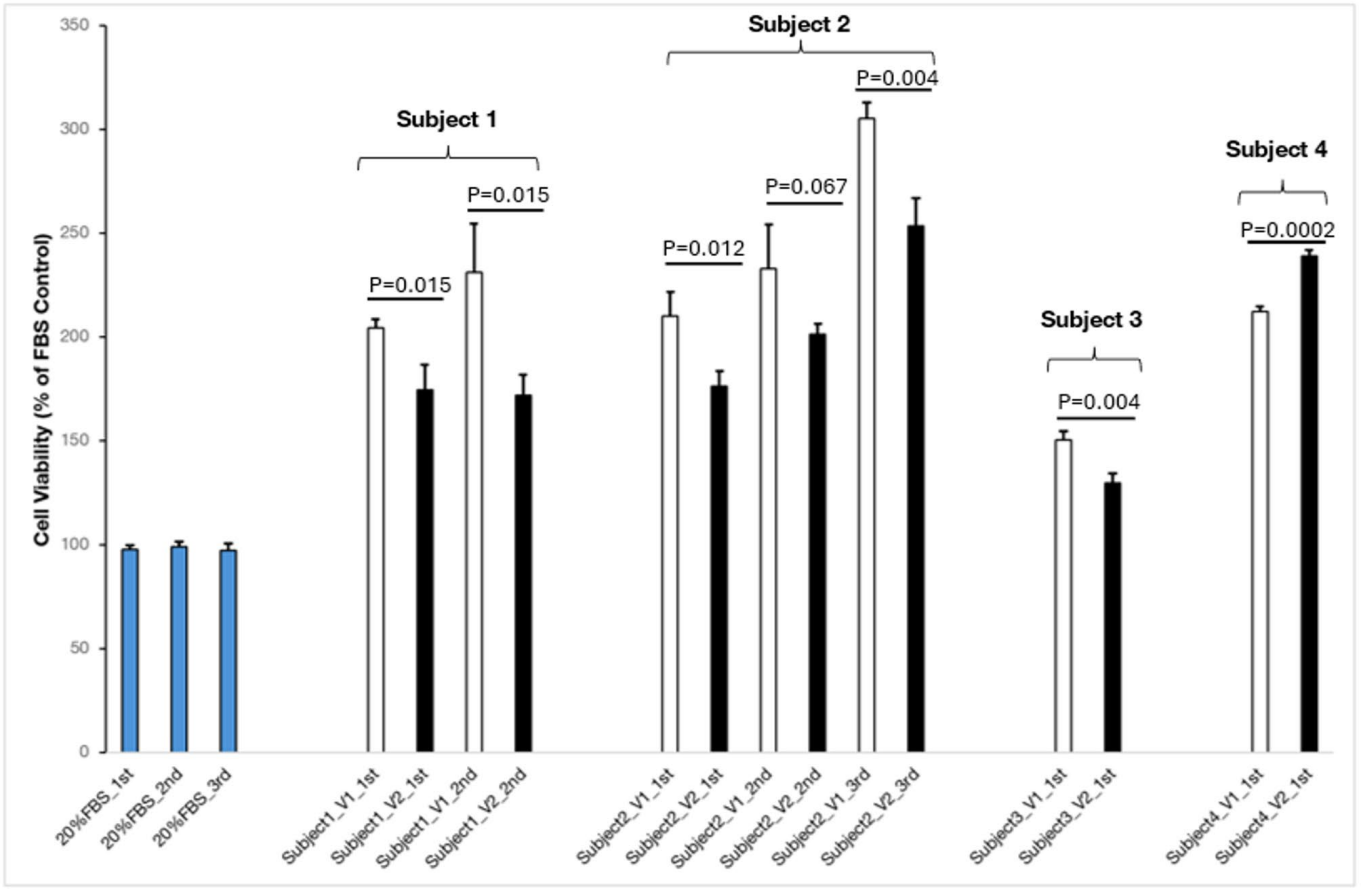
3-(4,5-dimethylthiazol-2-yl)−2,5-diphenyltetrazolium bromide (MTT) cell proliferation/viability assay on HepG2 cells treated with non-fasted (V1) and dry-fasted (V2) serum collected from subjects with metabolic syndrome and metabolic dysfunction-associated steatotic liver disease (MASLD) who fasted from dawn to dusk for 4 weeks (4-week DDDF). Compared to HepG2 liver tumor cells treated with human serum collected before 4-week DDDF (non-fasted serum), HepG2 cells that were treated with human serum collected at the end of 4-week DDDF (dry-fasted serum) showed significantly reduced cell proliferation in 3 out of the 4 subjects with metabolic syndrome and MASLD. A similar reduction in cell proliferation/viability was not observed in subject 4 with metabolic syndrome and MASLD. The effect of fetal bovine serum (FBS) on HepG2 cell proliferation/viability was much lower than that of non-fasted and dry-fasted human serum on HepG2 cell proliferation/viability

Compared to HepG2 cells treated with human serum collected before 4-week DDDF (non-fasted serum), HepG2 cells that were treated with human serum collected at the end of 4-week DDDF (dry-fasted serum) showed significantly reduced cell proliferation/viability in 3 out of 4 subjects with metabolic syndrome and MASLD (Fig. 2). In subject 1, on the first assay, mean HepG2 cell viability (% of fetal bovine serum control) for V1 vs. V2 was 204.50 (SD = 3.92) vs. 174.62 (SD = 12.00), *P* = 0.015, on the second assay, mean HepG2 cell viability for V1 vs. V2 was 231.38 (SD = 23.13) vs. 172.01 (SD = 9.69), *P* = 0.015; in subject 2, on the first assay, mean HepG2 cell viability for V1 vs. V2 was 210.23 (SD = 11.39) vs. 176.14 (SD = 7.29), *P* = 0.012, on the second assay, mean HepG2 cell viability for V1 vs. V2 was 232.88 (SD = 21.29) vs. 201.28 (SD = 5.08), *P* = 0.067, on the third assay, mean HepG2 cell viability for V1 vs. V2 was 305.16 (SD = 7.83) vs. 253.76 (SD = 13.31), *P* = 0.004; in subject 3, on the first assay, mean HepG2 cell viability for V1 vs. V2 was 150.64 (SD = 3.78) vs. 130.00 (SD = 4.43), *P* = 0.004. A similar reduction in cell viability was not observed in subject 4 with metabolic syndrome and MASLD: on the first assay, mean HepG2 cell viability for V1 vs. V2 was 212.47 (SD = 2.10) vs. 239.14 (SD = 2.61), *P* = 0.0002.

#### MTT assay in HepG2 cells treated with non-fasted and dry-fasted serum collected from healthy subjects

**Fig. 3:**
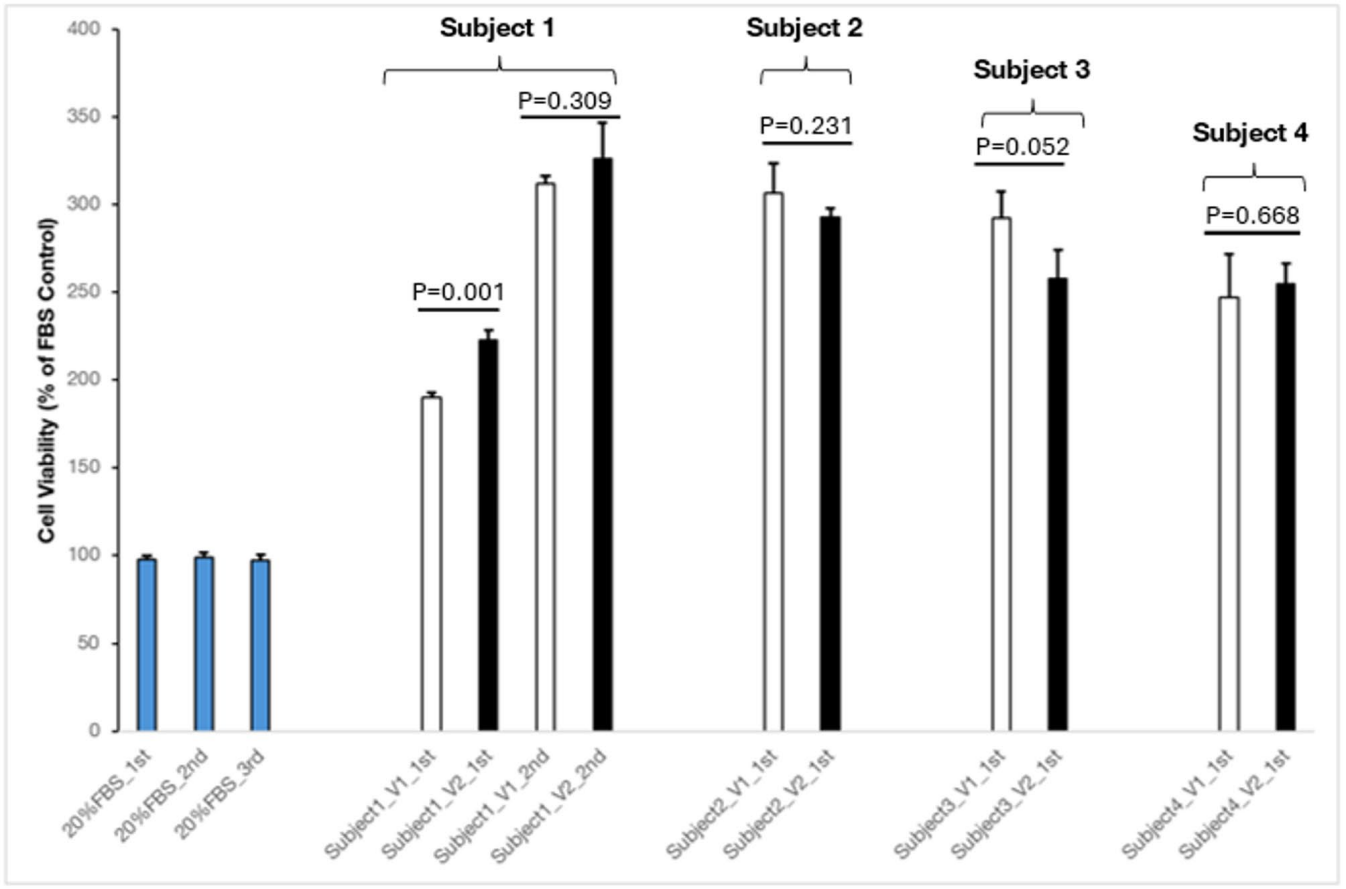
3-(4,5-dimethylthiazol-2-yl)−2,5-diphenyltetrazolium bromide (MTT) cell proliferation/viability assay on HepG2 cells treated with non-fasted (V1) and dry fasted (V2) serum collected from healthy subjects who fasted from dawn to dusk for 4 weeks (4-week DDDF). There was no significant decrease in cell proliferation in HepG2 cells that were treated with human serum collected at the end of 4-week DDDF (dry fasted serum) compared to HepG2 cells treated with human serum collected before 4-week DDDF (non-fasted serum). The effect of fetal bovine serum (FBS) on HepG2 cell proliferation/viability was much lower than that of non-fasted and dry-fasted human serum on HepG2 cell proliferation/viability

Compared to HepG2 cells treated with human serum collected before 4-week DDDF (non-fasted serum), HepG2 cells that were treated with human serum collected at the end of 4-week DDDF (dry-fasted serum) showed no significant decrease in cell proliferation/viability in healthy subjects (Fig. 3). In subject 1, on the first assay, V1 vs. V2 mean HepG2 cell viability (% of fetal bovine serum control) was 190.14 (SD = 2.69) vs. 222.95 (SD = 5.30), *P* = 0.001, on the second assay, V1 vs. V2 mean HepG2 cell viability was 311.94 (SD = 4.55) vs. 326.24 (SD = 20.80), *P* = 0.309; in subject 2, on the first assay, V1 vs. V2 mean HepG2 cell viability was 306.88 (SD = 16.68) vs. 292.69 (SD = 5.04), *P* = 0.231, in subject 3, on the first assay, V1 vs. V2 mean HepG2 cell viability was 292.58 (SD = 14.95) vs. 257.96 (SD = 16.02), *P* = 0.052, in subject 4, on the first assay, V1 vs. V2 mean HepG2 cell viability was 247.40 (SD = 24.27) vs. 254.63 (SD = 11.93), *P* = 0.668.

#### MTT assay in HepG2 cells treated with FBS

The effect of FBS on HepG2 cell proliferation/viability was much lower than that of non-fasted and dry-fasted human serum on HepG2 cell proliferation/viability (Figs. 2 and 3**)**. The mean cell viability in HepG2 cell culture treated with FBS was 97.72 (SD = 1.98) (first assay), 99.32 (SD = 2.22) (second assay), and 97.42 (SD = 3.18) (third assay).

### Human serum proteomics

#### Serum proteomics in subjects with metabolic syndrome and MASLD

The GPs that had greater than or equal to 1.5-fold mean paired change and a P-value of < 0.05 at the end of 4-week DDDF compared with the GP levels before 4-week DDDF are included in Table 1. There was an average 279 fold increase in CD248 molecule (CD248) (mean log2 fold = 8.124, *P* = 0.001), 2 fold increase in dipeptidyl peptidase 4 (DPP4) (mean log2 fold = 0.937, *P* = 0.027), 2 fold increase in lymphatic vessel endothelial hyaluronan receptor 1 (LYVE1) (mean log2 fold = 1.054, *P* = 0.029), 3 fold increase in LDL receptor-related protein 1 (LRP1) (mean log2 fold = 1.401, *P* = 0.031) GP levels compared with the GP levels before 4-week DDDF. There was a significant decrease in beta-2-microglobulin (BM2) (mean log2 fold= −0.977, *P* = 0.033) GP levels at the end of 4-week DDDF compared with the levels before 4-week DDDF. We included peptide spectrum match (PSM) of differentially expressed GPs in Additional file Table S1. The mean fold changes observed in all GPs are included in Additional file Table S2.

#### Serum proteomics in healthy subjects

The GPs that had greater than or equal to 1.5-fold mean paired change and a P-value of < 0.05 at the end of 4-week DDDF compared with the GP levels before 4-week DDDF are included in Table 2. There was an average 2-fold increase in charged multivesicular body protein 4 A (CHMP4 A) (mean log2 fold = 0.661, *P* = 0.045) GP levels compared with the GP levels before 4-week DDDF. There was a significant decrease in follistatin like 4 (FSTL4) (mean log 2 fold= −9.016, *P* = 0.0002), cystatin A (CSTA) (mean log2 fold= −12.325, *P* = 0.0003), cathepsin C (CTSC) (mean log2 fold= −8.542, *P* = 0.001), lipocalin 2 (LCN2) (mean log2 fold= −11.271, *P* = 0.002), fatty acid binding protein 5 (FABP5) (mean log2 fold=−12.127, *P* = 0.010), profilin 1 (PFN1) (mean log2 fold=−12.865, *P* = 0.012), tyrosine 3-monooxygenase/tryptophan 5-monooxygenase activation protein epsilon (YWHAE) (mean log2 fold= −9.769, *P* = 0.026), CD109 molecule (CD109) (mean log2 fold= −1.321, *P* = 0.039), CD93 molecule (CD93) (mean log2 fold= −2.567, *P* = 0.041), filamin A (FLNA) (mean log2 fold= −10.331, *P* = 0.0498) at the end of 4-week DDDF compared with the GP levels before 4-week DDDF. We included PSM of differentially expressed GPs in Additional file Table S3. The mean fold changes observed in all GPs are included in Additional file Table S4.

#### Prize-Collecting Steiner Forest (PCSF) algorithm analysis

**Fig. 4:**
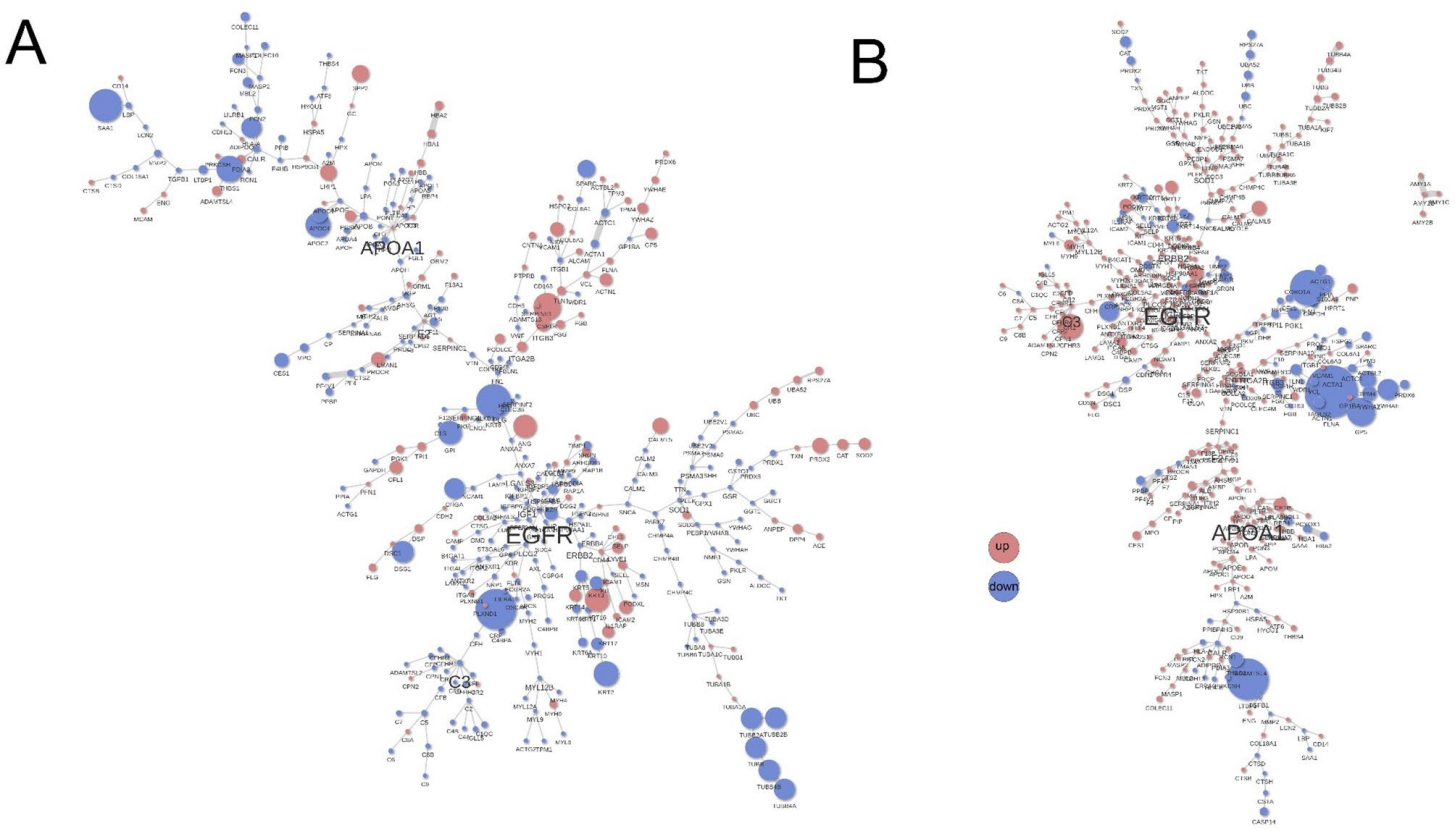
Prize-Collecting Steiner Forest (PCSF) algorithm analysis of serum gene protein products (GP) relative aduncity change in (A) subjects with metabolic syndrome and metabolic dysfunction-associated steatotic liver disease (MASLD) and (B) healthy subjects at the end of 4-week dawn-to-dusk dry fasting (DDDF) compared with the GP levels before 4-week DDDF. The resulting PCSF subnetwork revealed that proteins associated with the EGFR and APOA1 pathways were generally decreased in serum samples collected from subjects with metabolic syndrome and MASLD at the end of 4-week DDDF, compared with serum samples collecteed before 4-week DDDF (A). In contrast, EGFR and APOA1 node protein levels were elevated in dry-fasted serum compared with non-fasted serum from healthy individuals (B)

The analysis of the PCSF subnetwork showed that proteins associated with the EGFR and APOA1 pathways were generally decreased in serum samples collected from subjects with metabolic syndrome and MASLD at the end of 4-week DDDF compared with the serum collected before 4-week DDDF (Fig. 4A). In contrast, EGFR and APOA1 node protein levels were elevated in serum samples taken from healthy subjects at the end of 4-week DDDF compared with the serum collected before 4-week DDDF (Fig. 4B). 

## Discussion

We cultured HepG2 cells using non-fasted and dry-fasted human serum from two distinct groups of subjects and analyzed the serum proteome of the same individuals. This led to three critical observations: (1) Serum collected from most of the subjects with metabolic syndrome and MASLD at the end of 4-week DDDF significantly decreased cell proliferation/viability in HepG2 cells compared with the serum collected before 4-week DDDF; (2) serum collected from healthy subjects at the end of 4-week DDDF did not significantly decrease cell proliferation/viability in HepG2 cells; and (3) there was a differential gene expression (both increases and decreases) when comparing serum protein levels at the end of the 4-week DDDF with those measured before the 4-week DDDF in both groups.

The capability of dry-fasted serum collected from subjects with metabolic syndrome and MASLD to decrease cell proliferation in HepG2 liver tumor cell cultures could be due to the anti-tumorigenic proteome in the serum [11]. Our previous work showed that 4-week DDDF increased tumor-suppressor and DNA-repair proteins and decreased tumor-promoter proteins in the serum of subjects with metabolic syndrome [11]. We also showed that peripheral blood mononuclear cells (PBMC) collected from subjects with metabolic syndrome who performed 4-week DDDF showed an anti-inflammatory, anti-atherosclerotic, and anti-tumorigenic proteome [12]. Human serum conditioned by acute aerobic exercise was shown to have a similar anti-proliferative effect on LoVo human colon cancer cells [52]. A meta-analysis of 9 in vitro studies that evaluated the effect of exercise-conditioned human serum collected from 244 individuals showed that acute exercise-conditioned human serum significantly reduced proliferation in cancer cell lines, with the effect being more pronounced with acute high-intensity exercise-conditioned human serum [53]. These findings indicate that DDDF, mealtimes, and exercise impact cancer behavior. Human serum conditioned by 4-week DDDF combined with acute high-intensity exercise may substantially reduce cancer cell proliferation compared with their individual effects alone. Further studies are needed to confirm this hypothesis.

We observed a significant reduction in cell proliferation in HepG2 cells treated with dry-fasted serum collected from subjects with metabolic syndrome and MASLD. However, no similar reduction was seen in these cells when treated with dry-fasted serum from healthy subjects. This suggests that the impact of DDDF on subjects with metabolic syndrome may be more pronounced compared with its impact on healthy individuals. Consequently, it is important to include more healthy subjects in the study to confirm this effect. Additionally, the lack of a significant reduction in the growth of HepG2 cells treated with dry-fasted serum from healthy subjects may be attributed to certain substances or factors present in their serum. Therefore, a greater volume of dry-fasted serum may be necessary to mitigate the influence of these factors. Several reports showed that human serum had substances that control the mode (dense clumps vs. loose migratory structure) and cell growth rate in tissue cultures [54–57]. Different effects of human serum were observed by others in cell cultures based on distinct populations (healthy vs. pregnant vs. cancer) on tissue cultures [54–57]. Whereas normal human serum had a cell-proliferating effect, human serum collected from individuals with neoplasms, leukemia and pernicious anemia resulted in an inhibitory effect on bone marrow cultures [56]. Given the results of these previous reports [54–57] and our previous work that showed that 4-week DDDF induced an anti-tumorigenic proteome in healthy subjects [10], the suppressor effect of dry-fasted serum may be dose-dependent, and dose-response studies are needed.

In parallel to the reduction in cell proliferation/viability in HepG2 cells after treatment with dry-fasted serum, there were also significant changes in serum proteins in subjects with metabolic syndrome and MASLD at the end of 4-week DDDF compared with the serum proteins collected before 4-week DDDF. There was an average 279-fold increase in CD248 GP level at the end of 4-week DDDF compared with CD248 GP levels before 4-week DDDF. CD248, also known as endosialin and tumor endothelial marker 1 (TEM1) plays a significant role in hepatocellular tumorigenesis and physiological and pathological angiogenesis [58–60]. Mogler et al. [58] demonstrated that endosialin, expressed by hepatic stellate cells, plays a critical role in suppressing the proliferation of hepatocellular carcinoma. Silencing endosialin in these cells decreases their proliferation and accelerates the progression of hepatocellular carcinoma [58]. The study also found that endosialin-deficient mice exhibited higher levels of hepatic tumor cell proliferation and tumor burden than wild-type mice [58]. These findings strongly support the interpretation that 4-week DDDF-induced CD248 upregulation in subjects with metabolic syndrome and MASLD may represent an immune-regulatory response, contributing to the suppression of HepG2 liver cancer cell proliferation observed in our study. Although the upregulation of CD248 was associated with tumor angiogenesis and metastasis [59], the upregulation of CD248 in the dry fasted serum at the end of 4-week DDDF is likely due to physiological angiogenesis rather than pathological tumor angiogenesis observed in several cancers. In fact, it was shown that CD248, located in muscle pericytes, was crucial in sprouting angiogenesis during skeletal muscle remodeling, and CD248 knockout mice failed to have capillary sprouting [60]. These findings align with the findings of our previous study that showed upregulation of tropomyosin 4 one week after 30-day DDDF in healthy subjects [10]. Tropomyosin 4 was shown to have a significant role in skeletal muscle remodeling [61]. Further research is necessary to examine the effects of DDDF on skeletal muscle remodeling and development. This is particularly important because a TRE regimen that omitted breakfast and permitted study participants to consume water, black coffee and tea during the fasting period led to significant loss of lean mass [25]. 

Serum proteomic analysis showed that DPP4, also known as CD26, was upregulated at the end of 4-week DDDF. Upregulation of DDP4 is likely associated with the anti-tumorigenic effect of human dry-fasted serum. Patients with gastric cancer were shown to have significantly decreased serum CD26 (DDP4) levels compared to healthy controls [62]. The same study also showed that serum CD26 levels were lower in patients with HER2-positive tumors than those with HER2-negative tumors [62]. Similarly, serum CD26 levels in preoperative patients with colorectal carcinoma were shown to be significantly lower compared with the serum CD26 levels in healthy controls [63]. 

Using serum proteomic analysis, we found an average 2-fold increase in LYVE1 and a 3-fold increase in LRP1 GP levels at the end of 4-week DDDF compared with the GP levels before 4-week DDDF. LYVE-1, a scavenger hyaluronan receptor and CD44 homolog, is located in the lymph vessels and hepatic sinusoids [64]. Patients with metastatic lung cancer were shown to have lower serum LYVE-1 levels compared with patients with non-metastatic lung cancer, and the size of the primary tumor showed an inverse correlation with serum LYVE-1 levels [65]. The expression of LYVE-1 was also shown to be downregulated in cirrhosis and hepatocellular carcinoma [64, 66]; the downregulation of LYVE-1, the scavenger hyaluronan receptor, likely resulting in overexpression of hyaluronan and nuclear translocation of CD44-mediated pyruvate kinase M2 and thereby leading to the progression and poor prognosis in hepatocellular carcinoma [64, 66, 67]. Hyaluronan was shown to induce HepG2 liver cancer cell proliferation [67], which could also be related to the downregulation of LYVE-1. Similar to LYVE1, downregulation of LRP1 was also associated with invasiveness and poor prognosis in hepatocellular carcinoma [68]. Our results showing the upregulation of LYVE-1 and LRP1 in the serum after 4-week DDDF and reduction in HepG2 cell proliferation after treatment with dry-fasted serum align with the findings of these previous studies.

There are several human studies that reported anti-inflammatory benefits with dry fasting [69–72]. There are also murine studies that showed anti-tumorigenic effect with water fasting [9, 13]. Water fasting cycles lasting 48 to 60 h have been shown to delay tumor progression and enhance sensitivity to chemotherapy drugs in murine models of melanoma, neuroblastoma, and breast cancer [9]. In a murine osteosarcoma model, food deprivation during the active period produced a more significant anti-tumor effect compared to food deprivation during the inactive period and ad libitum feeding, and these outcomes were attributed to an improvement in the host’s ability to control the tumor or to alterations in the tumor’s circadian clock, or both [13]. 

The analysis of the PCSF subnetwork showed that proteins associated with the EGFR and APOA1 pathways were generally decreased in serum samples taken from subjects with metabolic syndrome and MASLD at the end of 4-week DDDF compared with the serum collected before 4-week DDDF (Fig. 4A). In contrast, EGFR and APOA1 node protein levels were elevated in serum samples taken from healthy subjects at the end of 4-week DDDF compared with the serum collected before 4-week DDDF (Fig. 4B). While the relationship between circulating EGFR and APOA1 levels and tumor cell growth remains complex and controversial, this differential alteration in EGFR and APOA1 levels may be associated with the observed inhibition of proliferation in the cultured HepG2 cells treated with these serum samples. For example, EGFR knockdown has been shown to reduce the proliferation and migration of HepG2 cells via Akt/GSK-3β/Snail signaling pathway [73]. The EGFR inhibition by erlotinib was found to overcome lenvatinib drug resistance in hepatocellular carcinoma by blocking the activation of the EGFR–STAT3–ABCB1 signaling pathway [74]. One particularly intriguing aspect is the observed downregulation of APOA1 in serum collected from subjects with metabolic syndrome and MASLD. The downregulation of APOA1 is linked to increased tumor progression in cancers such as basal-like breast cancer and hepatocellular carcinoma, whereas overexpression of APOA1 has shown anti-proliferative and pro-apoptotic effects [75–77]. These findings suggest that both EGFR and APOA1 play critical roles in maintaining proliferative potential. Further investigation is needed to clarify the relationship between serum-level alterations of these proteins and their effects on cancer cell proliferation.

To determine whether the effect of human non-fasted and dry-fasted serums differed from that of a fetal bovine serum on HepG2 cells, we treated HepG2 cells with both human serum and FBS. The influence of human serum on cell proliferation was significantly greater than that of FBS which served as a control under standard cell growth conditions. This effect was consistent regardless of whether the serum was derived from healthy individuals or those with metabolic syndrome and MASLD, as well as whether the subjects were non-fasted or dry-fasted. This suggests that human serum supplementation is much more favorable for the growth of human-derived HepG2 cells (Figs. 2 and 3). The behavioral differences between human and bovine serum in tissue cultures were previously reported [78, 79]. Our results suggest that human serum should be used instead of FBS in cell culture experiments to mimic human metabolism.

The limitations and challenges of our study are as follows: To our knowledge, this is the first study to test human serum obtained from subjects who underwent 4-week DDDF on HepG2 liver cancer cell viability. The MTT assay, a widely used and well-established method for assessing cell viability, was employed in this study to evaluate HepG2 cell proliferation. However, it primarily reflects mitochondrial activity rather than direct cell death, meaning that metabolic alterations could influence the observed effects independently of changes in cell viability.

Because there is a lack of prior research in this area, we were unable to determine the statistical power in advance; therefore, we classify our study as a pilot study. Additionally, because we collected samples from a limited number of subjects, we could not account for other factors such as age, gender, or lifestyle, which limits the scope of our research.

Based on an expected mean difference of 15% in cancer cell proliferation between serum samples collected before and at the end of 4-week DDDF, and with an estimated standard deviation of 20%, a two-sided paired t-test with 80% power and a significance level of 0.05 requires a minimum of 15 matched serum samples to detect a statistically significant effect. The sample size was estimated using G*Power 3.1, a validated and widely used statistical software for power analysis in biomedical research [80]. Despite recognizing the limitations of our data, this study serves as proof-of-concept. Further validation in larger, independent cohorts using antibody-based methods could enhance our findings.

Our study demonstrated that a 4-week DDDF alters the serum proteome, regardless of whether these changes are statistically significant at a P-value of < 0.05. There is a possibility that DDDF has initiated a series of complex molecular changes throughout the body. These changes may represent a continuum that is not adequately captured by evaluating serum samples at just a single time point after the fasting period concludes. To fully understand the anti-tumorigenic effects of DDDF, it may be necessary to conduct a more comprehensive analysis over multiple time intervals, allowing researchers to gain deeper insights into how these changes unfold and interact over time. Our observations are likely related to the Warburg effect that represents metabolic stress and the reprogramming of protein translation that could be fatal to malignant cells [14–16]. 

Furthermore, we only evaluated human non-fasted and dry-fasted serum in HepG2 cell cultures and did not conduct a dose-escalation study. Given these limitations, our preliminary results suggest that future research should address the following critical questions: (1) Does human dry-fasted serum have a similar suppressive effect on cancer cell lines other than HepG2? (2) Is the effect of human dry-fasted serum on suppressing liver tumor cell viability and invasiveness dose-dependent? (3) Does co-culturing liver cancer cell lines with human dry-fasted PBMC produce a more substantial inhibitory effect on cell viability and invasiveness than culturing liver cancer cell lines with dry-fasted serum alone? Addressing these questions may uncover new therapeutic implications for dry-fasted serum, plasma, or blood in cancer treatment, particularly since many cancer patients cannot undergo dry fasting due to chemotherapy-related side effects such as nausea, vomiting, and diarrhea.

There may be potential challenges in translating DDDF into clinical practice. One anticipated challenge is ensuring subject compliance with the DDDF regimen, similar to any type of fasting program. Participants must schedule their meals at dawn and dusk, abstaining from eating and drinking during the daytime to align their mealtimes, wake-up times, and sleep times with the natural cycle of dawn and dusk. This adjustment requires a strong commitment to self-discipline and willpower [81]. 

Additionally, because the fasting duration spans from dawn to dusk, it will vary from day to day and season to season. This variability is expected and is actually beneficial, as it can help precisely reset the circadian clock. In contrast, fasting with a predetermined, fixed number of hours would not align with the natural times of dawn and dusk and could disrupt the circadian rhythm.

## Conclusion

To our knowledge, this is the first study demonstrating dry-fasted serum collected from subjects with metabolic syndrome and MASLD reduced proliferation/viability of HepG2 liver cancer cells in vitro and showed changes in the proteome in vivo compared with non-fasted serum collected from the same subjects. Our findings suggest that 4-week DDDF might be an intervention to induce proteomic responses for the prevention and adjunct treatment of metabolic syndrome-induced cancers (e.g., liver, colorectal, pancreas, breast cancer).

**Table 1:** Differentially expressed gene protein products (GP) in serum collected from subjects with metabolic syndrome and metabolic-dysfunction associated steatotic liver (MASLD) who performed 4-week dawn-to-dusk dry fasting (DDDF)

Gene Symbol	Gene ID	Gene Name	Average Paired Log2 Fold Change*	Paired *P* Value
**GP Levels that Increased at the End of 4-Week DDDF Compared with the GP Levels Before 4-Week DDDF**				
CD248	57124	CD248 Molecule	8.124	0.001
DPP4	1803	Dipeptidyl Peptidase 4	0.937	0.027
LYVE1	10894	Lymphatic Vessel Endothelial Hyaluronan Receptor 1	1.054	0.029
LRP1	4035	LDL Receptor-Related Protein 1	1.401	0.031
**GP Levels that Decreased at the End of 4-Week DDDF Compared with the GP Levels Before 4-Week DDDF**				
B2M	567	Beta-2-Microglobulin	−0.977	0.033

* A positive mean paired log2 fold change indicates an increase, and negative mean paired log2 fold change indicates a decrease in the levels

**Table 2:** Differentially expressed gene protein products (GP) in serum collected from healthy subjects who performed 4-week dawn-to-dusk dry fasting (DDDF)

Gene Symbol	Gene ID	Gene Name	Average Paired Log2 Fold Change*	Paired *P* Value
**GP Levels that Increased at the End of 4-Week DDDF Compared with the GP Levels Before 4-Week DDDF**				
CHMP4A	29082	Charged Multivesicular Body Protein 4A	0.661	0.045
**GP Levels that Decreased at the End of 4-Week DDDF Compared with the GP Levels Before 4-Week DDDF**				
FSTL4	23105	Follistatin Like 4	−9.016	0.0002
CSTA	1475	Cystatin A	−12.325	0.0003
CTSC	1075	Cathepsin C	−8.542	0.001
LCN2	3934	Lipocalin 2	−11.271	0.002
FABP5	2171	Fatty Acid Binding Protein 5	−12.127	0.010
PFN1	5216	Profilin 1	−12.865	0.012
YWHAE	7531	Tyrosine 3-Monooxygenase/Tryptophan 5-Monooxygenase Activation Protein Epsilon	−9.769	0.026
CD109	135228	CD109 Molecule	−1.321	0.039
CD93	22918	CD93 Molecule	−2.567	0.041
FLNA	2316	Filamin A	−10.331	0.0498

* A positive mean paired log2 fold change indicates an increase, and negative mean paired log2 fold change indicates a decrease in the levels

## Supplementary Information

Additional file 1

Additional file 2

Additional file 3

Additional file 4

## Data Availability

Data Availability Statement: The raw data are not publicly available due to privacy and ethical restrictions.

## References

[1] Saklayen MG. The global epidemic of the metabolic syndrome. Curr Hypertens Rep. 2018;20:12. 29480368 10.1007/s11906-018-0812-z PMC5866840

[2] Younossi ZM, Golabi P, Paik JM, Henry A, Van Dongen C, Henry L. The global epidemiology of nonalcoholic fatty liver disease (NAFLD) and nonalcoholic steatohepatitis (NASH): a systematic review. Hepatology. 2023;77:1335–47. 36626630 10.1097/HEP.0000000000000004 PMC10026948

[3] Grundy SM, Cleeman JI, Daniels SR, Donato KA, Eckel RH, Franklin BA, Gordon DJ, Krauss RM, Savage PJ, Smith SC Jr., Spertus JA, Costa F, American Heart A, National Heart L, Blood I. Diagnosis and management of the metabolic syndrome: an American heart association/national heart, lung, and blood Institute scientific statement. Circulation. 2005;112:2735–52. 16157765 10.1161/CIRCULATIONAHA.105.169404

[4] Picchi A, Gao X, Belmadani S, Potter BJ, Focardi M, Chilian WM, Zhang C. Tumor necrosis factor-alpha induces endothelial dysfunction in the prediabetic metabolic syndrome. Circ Res. 2006;99:69–77. 16741160 10.1161/01.RES.0000229685.37402.80

[5] Vykoukal D, Davies MG. Vascular biology of metabolic syndrome. J Vasc Surg. 2011;54:819–31. 21439758 10.1016/j.jvs.2011.01.003 PMC3136643

[6] Radu F, Potcovaru CG, Salmen T, Filip PV, Pop C, Fierbinteanu-Braticievici C. The link between NAFLD and metabolic syndrome. Diagnostics 2023;13:614. 10.3390/diagnostics13040614 PMC9955701 36832102

[7] Esposito K, Chiodini P, Colao A, Lenzi A, Giugliano D. Metabolic syndrome and risk of cancer: a systematic review and meta-analysis. Diabetes Care. 2012;35:2402–11. 23093685 10.2337/dc12-0336 PMC3476894

[8] Welzel TM, Graubard BI, Zeuzem S, El-Serag HB, Davila JA, McGlynn KA. Metabolic syndrome increases the risk of primary liver cancer in the united States: a study in the SEER-Medicare database. Hepatology. 2011;54:463–71. 21538440 10.1002/hep.24397 PMC4141525

[9] Lee C, Raffaghello L, Brandhorst S, Safdie FM, Bianchi G, Martin-Montalvo A, Pistoia V, Wei M, Hwang S, Merlino A, Emionite L, de Cabo R, Longo VD. Fasting cycles retard growth of tumors and sensitize a range of cancer cell types to chemotherapy. Sci Transl Med. 2012;4:124ra127. 10.1126/scitranslmed.3003293 PMC3608686 22323820

[10] Mindikoglu AL, Abdulsada MM, Jain A, Choi JM, Jalal PK, Devaraj S, Mezzari MP, Petrosino JF, Opekun AR, Jung SY. Intermittent fasting from dawn to sunset for 30 consecutive days is associated with anticancer proteomic signature and upregulates key regulatory proteins of glucose and lipid metabolism, circadian clock, DNA repair, cytoskeleton remodeling, immune system and cognitive function in healthy subjects. J Proteom. 2020;217:103645. 10.1016/j.jprot.2020.103645 PMC7429999 31927066

[11] Mindikoglu AL, Abdulsada MM, Jain A, Jalal PK, Devaraj S, Wilhelm ZR, Opekun AR, Jung SY. Intermittent fasting from dawn to sunset for four consecutive weeks induces anticancer serum proteome response and improves metabolic syndrome. Sci Rep. 2020;10:18341. 33110154 10.1038/s41598-020-73767-w PMC7592042

[12] Mindikoglu AL, Park J, Opekun AR, Abdulsada MM, Wilhelm ZR, Jalal PK, Devaraj S, Jung SY. Dawn-to-dusk dry fasting induces anti-atherosclerotic, anti-inflammatory, and anti-tumorigenic proteome in peripheral blood mononuclear cells in subjects with metabolic syndrome. Metabol Open. 2022;16:100214. 36506940 10.1016/j.metop.2022.100214 PMC9731888

[13] Wu MW, Li XM, Xian LJ, Levi F. Effects of meal timing on tumor progression in mice. Life Sci. 2004;75:1181–93. 15219806 10.1016/j.lfs.2004.02.014

[14] de Cabo R, Mattson MP. Effects of intermittent fasting on health, aging, and disease. N Engl J Med. 2019;381:2541–51. 31881139 10.1056/NEJMra1905136

[15] Warburg O. The metabolism of carcinoma cells. J Cancer Res. 1925;9:148–63.

[16] Shaiken TE, Grimm SL, Siam M, Williams A, Rezaeian AH, Kraushaar D, Ricco E, Robertson MJ, Coarfa C, Jain A, Malovannaya A, Stossi F, Opekun AR, Price AP, Dubrulle J. Transcriptome, proteome, and protein synthesis within the intracellular cytomatrix. iScience. 2023;26:105965. 36824274 10.1016/j.isci.2023.105965 PMC9941065

[17] Damiola F, Le Minh N, Preitner N, Kornmann B, Fleury-Olela F, Schibler U. Restricted feeding uncouples circadian oscillators in peripheral tissues from the central pacemaker in the Suprachiasmatic nucleus. Genes Dev. 2000;14:2950–61. 11114885 10.1101/gad.183500 PMC317100

[18] Meijer JH, Groos GA, Rusak B. Luminance coding in a circadian pacemaker: the Suprachiasmatic nucleus of the rat and the hamster. Brain Res. 1986;382:109–18. 3768668 10.1016/0006-8993(86)90117-4

[19] Boege HL, Bhatti MZ, St-Onge MP. Circadian rhythms and meal timing: impact on energy balance and body weight. Curr Opin Biotechnol. 2021;70:1–6. 32998085 10.1016/j.copbio.2020.08.009 PMC7997809

[20] Witbracht M, Keim NL, Forester S, Widaman A, Laugero K. Female breakfast skippers display a disrupted cortisol rhythm and elevated blood pressure. Physiol Behav. 2015;140:215–21. 25545767 10.1016/j.physbeh.2014.12.044

[21] Nas A, Mirza N, Hagele F, Kahlhofer J, Keller J, Rising R, Kufer TA, Bosy-Westphal A. Impact of breakfast skipping compared with dinner skipping on regulation of energy balance and metabolic risk. Am J Clin Nutr. 2017;105:1351–61. 28490511 10.3945/ajcn.116.151332

[22] Smith KJ, Gall SL, McNaughton SA, Blizzard L, Dwyer T, Venn AJ. Skipping breakfast: longitudinal associations with cardiometabolic risk factors in the childhood determinants of adult health study. Am J Clin Nutr. 2010;92:1316–25. 20926520 10.3945/ajcn.2010.30101

[23] Qin LQ, Li J, Wang Y, Wang J, Xu JY, Kaneko T. The effects of nocturnal life on endocrine circadian patterns in healthy adults. Life Sci. 2003;73:2467–75. 12954455 10.1016/s0024-3205(03)00628-3

[24] Gu C, Brereton N, Schweitzer A, Cotter M, Duan D, Borsheim E, Wolfe RR, Pham LV, Polotsky VY, Jun JC. Metabolic effects of late dinner in healthy Volunteers-A randomized crossover clinical trial. J Clin Endocrinol Metab. 2020;105:2789–802. 32525525 10.1210/clinem/dgaa354 PMC7337187

[25] Lowe DA, Wu N, Rohdin-Bibby L, Moore AH, Kelly N, Liu YE, Philip E, Vittinghoff E, Heymsfield SB, Olgin JE, Shepherd JA, Weiss EJ. Effects of Time-Restricted eating on weight loss and other metabolic parameters in women and men with overweight and obesity: the TREAT randomized clinical trial. JAMA Intern Med. 2020;180:1491–9. 32986097 10.1001/jamainternmed.2020.4153 PMC7522780

[26] Dunning A. The Phoenix Protocol Dry Fasting for Rapid Healing and Radical Life Extension: Functional Immortality. Independently Published, 2020.

[27] Filonov SI. 20 Questions & Answers About Dry Fasting: A Complete Guide to Dry Fasting. Siberika Publishing, 2019.

[28] Geelen G, Greenleaf JE, Keil LC. Drinking-induced plasma vasopressin and norepinephrine changes in dehydrated humans. J Clin Endocrinol Metab. 1996;81:2131–5. 8964840 10.1210/jcem.81.6.8964840

[29] Palazzo AJ, Malik KU, Weis MT. Vasopressin stimulates the mobilization and metabolism of triacylglycerol in perfused rabbit hearts. Am J Physiol. 1991;260:H604–612. 1996703 10.1152/ajpheart.1991.260.2.H604

[30] Labrie F, Giguere V, Proulx L, Lefevre G. Interactions between CRF, epinephrine, vasopressin and glucocorticoids in the control of ACTH secretion. J Steroid Biochem. 1984;20:153–60. 6323861 10.1016/0022-4731(84)90202-4

[31] Valenta LJ, Elias AN, Eisenberg H. ACTH stimulation of adrenal epinephrine and norepinephrine release. Horm Res. 1986;23:16–20. 3000912 10.1159/000180283

[32] Graca FA, Goncalves DA, Silveira WA, Lira EC, Chaves VE, Zanon NM, Garofalo MA, Kettelhut IC, Navegantes LC. Epinephrine depletion exacerbates the fasting-induced protein breakdown in fast-twitch skeletal muscles. Am J Physiol Endocrinol Metab. 2013;305:E1483–1494. 24169047 10.1152/ajpendo.00267.2013

[33] Pedersen SB, Bak JF, Holck P, Schmitz O, Richelsen B. Epinephrine stimulates human muscle lipoprotein lipase activity in vivo. Metabolism. 1999;48:461–4. 10206438 10.1016/s0026-0495(99)90104-x

[34] Hillier KAW, Longworth ZL, Vatanparast H. Healthcare professionals knowledge, attitude, practices, and perspectives providing care to Muslims in Western countries who fast during ramadan: a scoping review. Appl Physiol Nutr Metab. 2024;49:415–27. 38128071 10.1139/apnm-2023-0462

[35] Abdalla AH, Shaheen FA, Rassoul Z, Owda AK, Popovich WF, Mousa DH, al-Hawas F, al-Sulaiman MH, al-Khader AA. Effect of ramadan fasting on moslem kidney transplant recipients. Am J Nephrol. 1998;18:101–4. 9569950 10.1159/000013316

[36] Al Lami Z, Kurtca M, Atique MU, Opekun AR, Siam MS, Jalal PK, Najafi B, Devaraj S, Mindikoglu AL. Dawn-to-dusk dry fasting decreases Circulating inflammatory cytokines in subjects with increased body mass index. Metabol Open. 2024;21:100274. 38455231 10.1016/j.metop.2024.100274 PMC10918425

[37] Cheah SH, Ch’ng SL, Husain R, Duncan MT. Effects of fasting during ramadan on urinary excretion in Malaysian Muslims. Br J Nutr. 1990;63:329–37. 2334668 10.1079/bjn19900119

[38] Koppold-Liebscher DA, Klatte C, Demmrich S, Schwarz J, Kandil FI, Steckhan N, Ring R, Kessler CS, Jeitler M, Koller B, Ananthasubramaniam B, Eisenmann C, Mahler A, Boschmann M, Kramer A, Michalsen A. Effects of daytime dry fasting on hydration, glucose metabolism and circadian phase: A prospective exploratory cohort study in Baha’i volunteers. Front Nutr. 2021;8:662310. 34395487 10.3389/fnut.2021.662310 PMC8358295

[39] Leiper JB, Molla AM, Molla AM. Effects on health of fluid restriction during fasting in ramadan. Eur J Clin Nutr. 2003;57(Suppl 2):S30–38. 14681711 10.1038/sj.ejcn.1601899

[40] Mellanby K. Metabolic water and desiccation. Nature. 1942;150:21–21.

[41] Edney EB. Metabolic water. In: Edney EB, editor. Water balance in land arthropods. Berlin, Heidelberg: Springer Berlin Heidelberg; 1977. pp. 189–95.

[42] Litwack G: Chapter 3 - Introductory Discussion on Water, pH, Buffers, and General Features of Receptors, Channels, and Pumps. In: Litwack G, ed. Human Biochemistry (Second Edition). Boston: Academic Press, 2022; 45-70.

[43] Crook MA, Hally V, Panteli JV. The importance of the refeeding syndrome. Nutrition. 2001;17:632–7. 11448586 10.1016/s0899-9007(01)00542-1

[44] Aden DP, Fogel A, Plotkin S, Damjanov I, Knowles BB. Controlled synthesis of HBsAg in a differentiated human liver carcinoma-derived cell line. Nature. 1979;282:615–6. 233137 10.1038/282615a0

[45] HEPG2. Available at https://www.atcc.org/products/hb-8065. Accessed on September 10, 2023.

[46] Lopez-Terrada D, Cheung SW, Finegold MJ, Knowles BB. Hep G2 is a hepatoblastoma-derived cell line. Hum Pathol. 2009;40:1512–5. 19751877 10.1016/j.humpath.2009.07.003

[47] Rappsilber J, Ishihama Y, Mann M. Stop and go extraction tips for matrix-assisted laser desorption/ionization, nanoelectrospray, and LC/MS sample pretreatment in proteomics. Anal Chem. 2003;75:663–70. 12585499 10.1021/ac026117i

[48] Saltzman AB, Leng M, Bhatt B, Singh P, Chan DW, Dobrolecki L, Chandrasekaran H, Choi JM, Jain A, Jung SY, Lewis MT, Ellis MJ, Malovannaya A. GpGrouper: A peptide grouping algorithm for Gene-Centric inference and quantitation of Bottom-Up proteomics data. Mol Cell Proteom. 2018;17:2270–83. 10.1074/mcp.TIR118.000850 PMC6210220 30093420

[49] SAS software. http://www.SAS.com/. The data analysis for this paper was generated using SAS software, Version 9.4 of the SAS system for Windows. Copyright © 2020 SAS Institute Inc. SAS and all other SAS Institute Inc. product or service names are registered trademarks or trademarks of SAS Institute Inc, Cary, NC, USA.

[50] Akhmedov M, Martinelli A, Geiger R, Kwee I. Omics playground: a comprehensive self-service platform for visualization, analytics and exploration of big omics data. NAR Genom Bioinform. 2019;2:lqz019. 33575569 10.1093/nargab/lqz019 PMC7671354

[51] Tuncbag N, Braunstein A, Pagnani A, Huang SS, Chayes J, Borgs C, Zecchina R, Fraenkel E. Simultaneous reconstruction of multiple signaling pathways via the prize-collecting steiner forest problem. J Comput Biol. 2013;20:124–36. 23383998 10.1089/cmb.2012.0092 PMC3576906

[52] Orange ST, Jordan AR, Odell A, Kavanagh O, Hicks KM, Eaglen T, Todryk S, Saxton JM. Acute aerobic exercise-conditioned serum reduces colon cancer cell proliferation in vitro through interleukin-6-induced regulation of DNA damage. Int J Cancer. 2022;151:265–74. 35213038 10.1002/ijc.33982 PMC9314683

[53] Soares CM, Teixeira AM, Sarmento H, Silva FM, Rusenhack MC, Furmann M, Nobre PR, Fachada MA, Urbano AM, Ferreira JP. Effect of exercise-conditioned human serum on the viability of cancer cell cultures: A systematic review and meta-analysis. Exerc Immunol Rev. 2021;27:24–41. 33965899

[54] Penttinen K, Saxen E. Growth-controlling action of human serum in cell culture. Nature. 1959;184(Suppl 20):1570–1. 10.1038/1841570b0 14431650

[55] Norris ER, Majnarich JJ. Cell proliferation accelerating and inhibiting substances in normal and cancer blood and urine. Proc Soc Exp Biol Med. 1949;70:229–34. 18112773 10.3181/00379727-70-16884

[56] Norris ER, Majnarich JJ. Effect of normal blood serum and blood serum from neoplastic disease on cell proliferation in bone marrow cultures. Am J Physiol. 1948;153:483–7. 18882184 10.1152/ajplegacy.1948.153.3.483

[57] Saxen E, Penttinen K. Differences in the effect of individual human Sera on cell cultures. J Natl Cancer Inst. 1965;35:67–73. 5889796 10.1093/jnci/35.1.67

[58] Mogler C, König C, Wieland M, Runge A, Besemfelder E, Komljenovic D, Longerich T, Schirmacher P, Augustin HG. Hepatic stellate cells limit hepatocellular carcinoma progression through the orphan receptor endosialin. EMBO Mol Med. 2017;9:741–9. 28373218 10.15252/emmm.201607222 PMC5452049

[59] Nanda A, Karim B, Peng Z, Liu G, Qiu W, Gan C, Vogelstein B, St Croix B, Kinzler KW, Huso DL. Tumor endothelial marker 1 (Tem1) functions in the growth and progression of abdominal tumors. Proc Natl Acad Sci U S A. 2006;103:3351–6. 16492758 10.1073/pnas.0511306103 PMC1413931

[60] Naylor AJ, McGettrick HM, Maynard WD, May P, Barone F, Croft AP, Egginton S, Buckley CD. A differential role for CD248 (Endosialin) in PDGF-mediated skeletal muscle angiogenesis. PLoS ONE. 2014;9:e107146. 25243742 10.1371/journal.pone.0107146 PMC4171374

[61] Vlahovich N, Schevzov G, Nair-Shaliker V, Ilkovski B, Artap ST, Joya JE, Kee AJ, North KN, Gunning PW, Hardeman EC. Tropomyosin 4 defines novel filaments in skeletal muscle associated with muscle remodelling/regeneration in normal and diseased muscle. Cell Motil Cytoskeleton. 2008;65:73–85. 17968984 10.1002/cm.20245

[62] Boccardi V, Marano L, Rossetti RR, Rizzo MR, di Martino N, Paolisso G. Serum CD26 levels in patients with gastric cancer: a novel potential diagnostic marker. BMC Cancer. 2015;15:703. 26471376 10.1186/s12885-015-1757-0 PMC4608357

[63] Cordero OJ, Ayude D, Nogueira M, Rodriguez-Berrocal FJ, de la Cadena MP. Preoperative serum CD26 levels: diagnostic efficiency and predictive value for colorectal cancer. Br J Cancer. 2000;83:1139–46. 11027426 10.1054/bjoc.2000.1410 PMC2363587

[64] Mouta Carreira C, Nasser SM, di Tomaso E, Padera TP, Boucher Y, Tomarev SI, Jain RK. LYVE-1 is not restricted to the lymph vessels: expression in normal liver blood sinusoids and down-regulation in human liver cancer and cirrhosis. Cancer Res. 2001;61:8079–84. 11719431

[65] Nunomiya K, Shibata Y, Abe S, Inoue S, Igarashi A, Yamauchi K, Kimura T, Aida Y, Nemoto T, Sato M, Kishi H, Nakano H, Sato K, Kubota I. Relationship between serum level of lymphatic vessel endothelial hyaluronan Receptor-1 and prognosis in patients with lung Cancer. J Cancer. 2014;5:242–7. 24665348 10.7150/jca.8486 PMC3963081

[66] Kitagawa K, Nakajima G, Kuramochi H, Ariizumi SI, Yamamoto M. Lymphatic vessel endothelial hyaluronan receptor-1 is a novel prognostic indicator for human hepatocellular carcinoma. Mol Clin Oncol. 2013;1:1039–48. 24649290 10.3892/mco.2013.167 PMC3915697

[67] Li JH, Wang YC, Qin CD, Yao RR, Zhang R, Wang Y, Xie XY, Zhang L, Wang YH, Ren ZG. Over expression of hyaluronan promotes progression of HCC via CD44-mediated pyruvate kinase M2 nuclear translocation. Am J Cancer Res. 2016;6:509–21. 27186420 PMC4859677

[68] Huang XY, Shi GM, Devbhandari RP, Ke AW, Wang Y, Wang XY, Wang Z, Shi YH, Xiao YS, Ding ZB, Dai Z, Xu Y, Jia WP, Tang ZY, Fan J, Zhou J. Low level of low-density lipoprotein receptor-related protein 1 predicts an unfavorable prognosis of hepatocellular carcinoma after curative resection. PLoS ONE. 2012;7:e32775. 22427881 10.1371/journal.pone.0032775 PMC3299691

[69] Papagiannopoulos-Vatopaidinos IE, Papagiannopoulou M, Sideris V. Dry fasting physiology: responses to hypovolemia and hypertonicity. Complement Med Res. 2020;27:242–51. 31958788 10.1159/000505201

[70] Faris MAE, Madkour MI, Obaideen AK, Dalah EZ, Hasan HA, Radwan H, Jahrami HA, Hamdy O, Mohammad MG. Effect of ramadan diurnal fasting on visceral adiposity and serum adipokines in overweight and obese individuals. Diabetes Res Clin Pract. 2019;153:166–75. 31150725 10.1016/j.diabres.2019.05.023

[71] Faris MA, Kacimi S, Al-Kurd RA, Fararjeh MA, Bustanji YK, Mohammad MK, Salem ML. Intermittent fasting during ramadan attenuates Proinflammatory cytokines and immune cells in healthy subjects. Nutr Res. 2012;32:947–55. 23244540 10.1016/j.nutres.2012.06.021

[72] Zouhal H, Bagheri R, Ashtary-Larky D, Wong A, Triki R, Hackney AC, Laher I, Abderrahman AB. Effects of ramadan intermittent fasting on inflammatory and biochemical biomarkers in males with obesity. Physiol Behav. 2020;225:113090. 32710888 10.1016/j.physbeh.2020.113090

[73] Gao J, Huo Z, Song X, Shao Q, Ren W, Huang X, Zhou S, Tang X. EGFR mediates epithelial–mesenchymal transition through the Akt/GSK-3β/Snail signaling pathway to promote liver cancer proliferation and migration. Oncol Lett. 2024;27:59. 38192662 10.3892/ol.2023.14192 PMC10773224

[74] Hu B, Zou T, Qin W, Shen X, Su Y, Li J, Chen Y, Zhang Z, Sun H, Zheng Y, Wang CQ, Wang Z, Li TE, Wang S, Zhu L, Wang X, Fu Y, Ren X, Dong Q, Qin LX. Inhibition of EGFR overcomes acquired lenvatinib resistance driven by STAT3-ABCB1 signaling in hepatocellular carcinoma. Cancer Res. 2022;82:3845–57. 36066408 10.1158/0008-5472.CAN-21-4140 PMC9574378

[75] Wang C, Chen S, Zhang R, Chen M, Yang X, He Y, Shangguan Z, Mao Q, Zhang Z, Ying S. Apolipoprotein A-1 downregulation promotes basal-like breast cancer cell proliferation and migration associated with DNA methylation. Oncol Lett. 2024;28:295. 38737975 10.3892/ol.2024.14428 PMC11082839

[76] Ma XL, Gao XH, Gong ZJ, Wu J, Tian L, Zhang CY, Zhou Y, Sun YF, Hu B, Qiu SJ, Zhou J, Fan J, Guo W, Yang XR. Apolipoprotein A1: a novel serum biomarker for predicting the prognosis of hepatocellular carcinoma after curative resection. Oncotarget. 2016;7:70654–68. 27683106 10.18632/oncotarget.12203 PMC5342581

[77] Wang Y, Chen S, Xiao X, Yang F, Wang J, Zong H, Gao Y, Huang C, Xu X, Fang M, Zhang X, Gao C. Impact of Apolipoprotein A1 on tumor immune microenvironment, clinical prognosis and genomic landscape in hepatocellular carcinoma. Precis Clin Med. 2023;6:pbad021. 38025972 10.1093/pcmedi/pbad021 PMC10680024

[78] Heger JI, Froehlich K, Pastuschek J, Schmidt A, Baer C, Mrowka R, Backsch C, Schleussner E, Markert UR, Schmidt A. Human serum alters cell culture behavior and improves spheroid formation in comparison to fetal bovine serum. Exp Cell Res. 2018;365:57–65. 29476836 10.1016/j.yexcr.2018.02.017

[79] Steenbergen R, Oti M, Ter Horst R, Tat W, Neufeldt C, Belovodskiy A, Chua TT, Cho WJ, Joyce M, Dutilh BE, Tyrrell DL. Establishing normal metabolism and differentiation in hepatocellular carcinoma cells by culturing in adult human serum. Sci Rep. 2018;8:11685. 30076349 10.1038/s41598-018-29763-2 PMC6076254

[80] Faul F, Erdfelder E, Buchner A, Lang AG. Statistical power analyses using G*Power 3.1: tests for correlation and regression analyses. Behav Res Methods. 2009;41:1149–60. 19897823 10.3758/BRM.41.4.1149

[81] Bhatti SI, Mindikoglu AL. The impact of dawn to sunset fasting on immune system and its clinical significance in COVID-19 pandemic. Metabol Open. 2022;13:100162. 34977523 10.1016/j.metop.2021.100162 PMC8713419

