# Supplementary material for: Decreased proliferation of HepG2 liver cancer cells in vitro and exhibited proteomic changes in vivo in subjects with metabolic syndrome and metabolic dysfunction-associated steatotic liver disease who performed four-week dawn-to-dusk dry fasting
Source: Clin Proteomics. 2025 Jun 24;22:25. doi: 10.1186/s12014-025-09547-3 (PMC12186377; doi:10.1186/s12014-025-09547-3)
Supplement: Supplementary file 1 — Additional file 1 [file 12014_2025_9547_MOESM1_ESM.docx]

**Supplementary Table 1.** Peptide Spectrum Match (PSM) of Differentially Expressed Gene Protein Products (GP) in Serum Collected from Subjects with Metabolic Syndrome and Metabolic-Dysfunction Associated Steatotic Liver (MASLD) Who Performed 4-Week Dawn-to-Dusk Dry Fasting (DDDF)

|  |  | **Before 4-Week DDDF** | | | | **At the End of 4-Week DDDF** | | | |
| --- | --- | --- | --- | --- | --- | --- | --- | --- | --- |
| **Gene ID** | **Gene Symbol** | **Subject 1** | **Subject 2** | **Subject 3** | **Subject 4** | **Subject 1** | **Subject 2** | **Subject 3** | **Subject 4** |
| 57124 | CD248 | ND | ND | ND | ND | 1 | 2 | 1 | ND |
| 1803 | DPP4 | 3 | 2 | 2 | ND | 5 | 2 | 3 | 2 |
| 10894 | LYVE1 | 4 | 4 | 5 | ND | 10 | 9 | 8 | 9 |
| 4035 | LRP1 | 4 | 3 | 3 | 1 | 7 | 2 | 6 | 4 |
| 567 | B2M | 8 | 8 | 7 | 1 | 6 | 6 | 6 | 5 |
