# Supplementary material for: Decreased proliferation of HepG2 liver cancer cells in vitro and exhibited proteomic changes in vivo in subjects with metabolic syndrome and metabolic dysfunction-associated steatotic liver disease who performed four-week dawn-to-dusk dry fasting
Source: Clin Proteomics. 2025 Jun 24;22:25. doi: 10.1186/s12014-025-09547-3 (PMC12186377; doi:10.1186/s12014-025-09547-3)
Supplement: Supplementary file 2 — Additional file 2 [file 12014_2025_9547_MOESM2_ESM.docx]

| **Supplementary Table S2**. Serum Gene Protein Product (GP) Levels that Increased or Decreased at the End of 4-Week Dawn-to-Dusk Dry Fasting (V2) Compared with the GP Levels Before 4-Week Dawn-to-Dusk Dry Fasting (V1) in Subjects with Metabolic Syndrome and Metabolic-Dysfunction Associated Steatotic Liver (MASLD) | | | |
| --- | --- | --- | --- |
| **Gene Symbol** | **Gene ID** | **Average Paired Log2 Fold Change (V2/V1)** | **Paired P Value** |
| CD248 | 57124 | 8.124 | 0.001 |
| DPP4 | 1803 | 0.937 | 0.027 |
| LYVE1 | 10894 | 1.054 | 0.029 |
| LRP1 | 4035 | 1.401 | 0.031 |
| B2M | 567 | -0.977 | 0.033 |
| PI16 | 221476 | 0.314 | 0.016 |
| TNXB | 7148 | 0.342 | 0.016 |
| KNG1 | 3827 | -0.220 | 0.016 |
| CHMP4A | 29082 | -0.293 | 0.022 |
| AHSG | 197 | -0.383 | 0.025 |
| HSPA5 | 3309 | 0.457 | 0.035 |
| IGFBP2 | 3485 | 0.554 | 0.046 |
| CACNA2D1 | 781 | 0.901 | 0.063 |
| APOC3 | 345 | -0.389 | 0.079 |
| C7 | 730 | -0.303 | 0.089 |
| PROS1 | 5627 | -0.170 | 0.104 |
| C1S | 716 | -0.209 | 0.111 |
| LGALS3BP | 3959 | 0.262 | 0.111 |
| HSP90B1 | 7184 | 0.494 | 0.115 |
| CFH | 3075 | -0.159 | 0.118 |
| SELL | 6402 | 0.861 | 0.121 |
| PCYOX1 | 51449 | 0.224 | 0.121 |
| PGLYRP1 | 8993 | 7.153 | 0.130 |
| TPI1 | 7167 | 7.304 | 0.131 |
| MBL2 | 4153 | -0.727 | 0.136 |
| AZGP1 | 563 | -0.114 | 0.142 |
| S100A9 | 6280 | 0.851 | 0.144 |
| ADAMTSL4 | 54507 | 8.434 | 0.145 |
| ICAM2 | 3384 | 1.220 | 0.158 |
| FSTL1 | 11167 | -6.448 | 0.158 |
| CRTAC1 | 55118 | 1.283 | 0.161 |
| CHL1 | 10752 | 0.560 | 0.166 |
| APOB | 338 | -0.507 | 0.169 |
| SPP2 | 6694 | 1.444 | 0.170 |
| IGLL5 | 100423062 | -0.307 | 0.170 |
| CFP | 5199 | -0.281 | 0.171 |
| NAGLU | 4669 | 7.238 | 0.172 |
| MMRN1 | 22915 | -0.762 | 0.176 |
| CP | 1356 | -0.181 | 0.177 |
| APOC2 | 344 | -1.581 | 0.178 |
| APOC4 | 346 | -1.050 | 0.179 |
| HTRA1 | 5654 | 5.601 | 0.184 |
| APOA5 | 116519 | 5.816 | 0.184 |
| ASGR2 | 433 | 8.589 | 0.184 |
| BLMH | 642 | 9.457 | 0.184 |
| NRCAM | 4897 | 4.658 | 0.184 |
| AIM1L | 55057 | -8.072 | 0.184 |
| C4B_2 | 100293534 | -0.375 | 0.184 |
| HSD11B1L | 374875 | 7.911 | 0.185 |
| LOC110384692 | 110384692 | -0.389 | 0.185 |
| FAM20C | 56975 | -6.211 | 0.186 |
| PRKCSH | 5589 | -6.323 | 0.187 |
| CNTN1 | 1272 | 0.742 | 0.187 |
| COL1A2 | 1278 | -3.913 | 0.188 |
| PKM | 5315 | 5.895 | 0.188 |
| MEGF8 | 1954 | -4.428 | 0.189 |
| MSN | 4478 | -5.916 | 0.189 |
| SAA2 | 6289 | -9.538 | 0.190 |
| C1orf56 | 54964 | 0.775 | 0.191 |
| FGB | 2244 | 7.431 | 0.191 |
| PRDX6 | 9588 | 6.515 | 0.192 |
| ADGRG6 | 57211 | 5.544 | 0.193 |
| CTBS | 1486 | 1.168 | 0.196 |
| KLKB1 | 3818 | -0.385 | 0.197 |
| TTR | 7276 | 0.347 | 0.200 |
| PHF21A | 51317 | 9.159 | 0.202 |
| RTN4RL2 | 349667 | 6.318 | 0.207 |
| KRT8 | 3856 | -1.504 | 0.207 |
| CTSD | 1509 | 7.127 | 0.208 |
| CFI | 3426 | -0.223 | 0.209 |
| CRISP3 | 10321 | 7.526 | 0.211 |
| HPR | 3250 | -0.469 | 0.213 |
| ITIH1 | 3697 | -0.263 | 0.214 |
| PPIB | 5479 | 5.691 | 0.215 |
| ROBO4 | 54538 | 5.127 | 0.215 |
| CSTA | 1475 | 8.710 | 0.222 |
| DSP | 1832 | 2.711 | 0.223 |
| LYZ | 4069 | -0.150 | 0.223 |
| SHBG | 6462 | 0.391 | 0.224 |
| CALML5 | 51806 | 6.367 | 0.225 |
| DBH | 1621 | -0.710 | 0.230 |
| SAA1 | 6288 | -6.830 | 0.232 |
| PROCR | 10544 | 1.266 | 0.243 |
| BST1 | 683 | 4.684 | 0.244 |
| GLIPR2 | 152007 | -7.413 | 0.245 |
| GPLD1 | 2822 | 0.477 | 0.246 |
| CDH6 | 1004 | 4.151 | 0.251 |
| PLG | 5340 | -0.206 | 0.252 |
| C6 | 729 | -0.174 | 0.255 |
| SERPINA11 | 256394 | 1.412 | 0.255 |
| PLXNB1 | 5364 | -2.749 | 0.257 |
| FABP5 | 2171 | 4.498 | 0.261 |
| SLC38A10 | 124565 | 3.740 | 0.261 |
| CDH2 | 1000 | 0.204 | 0.264 |
| VASN | 114990 | 0.089 | 0.265 |
| PTPRJ | 5795 | 4.523 | 0.269 |
| KRT2 | 3849 | -1.587 | 0.269 |
| TPM4 | 7171 | 0.872 | 0.274 |
| KRT4 | 3851 | -5.992 | 0.279 |
| SPARC | 6678 | -1.217 | 0.280 |
| F5 | 2153 | -0.398 | 0.283 |
| MB | 4151 | 4.779 | 0.284 |
| F13B | 2165 | -0.332 | 0.288 |
| ACE | 1636 | 4.482 | 0.291 |
| THBS1 | 7057 | -1.647 | 0.295 |
| MCAM | 4162 | 0.571 | 0.302 |
| IGFBP6 | 3489 | -0.271 | 0.305 |
| MGP | 4256 | 0.485 | 0.305 |
| SOD2 | 6648 | 3.559 | 0.311 |
| KRT16 | 3868 | 1.809 | 0.311 |
| PRG4 | 10216 | 0.490 | 0.312 |
| KRT14 | 3861 | 1.139 | 0.314 |
| DSC1 | 1823 | 3.503 | 0.316 |
| DMKN | 93099 | -3.520 | 0.318 |
| CD93 | 22918 | 4.759 | 0.321 |
| HEG1 | 57493 | 0.216 | 0.322 |
| SPARCL1 | 8404 | -0.212 | 0.323 |
| CFL1 | 1072 | 4.984 | 0.324 |
| PRDX2 | 7001 | 5.268 | 0.325 |
| RARRES2 | 5919 | 5.693 | 0.326 |
| SERPINE1 | 5054 | -3.609 | 0.328 |
| ITGB3 | 3690 | 3.666 | 0.332 |
| KRT6B | 3854 | 4.906 | 0.343 |
| C3 | 718 | -0.320 | 0.344 |
| CFHR1 | 3078 | 0.078 | 0.345 |
| SOD3 | 6649 | 0.814 | 0.348 |
| SELENBP1 | 8991 | 3.488 | 0.348 |
| F11 | 2160 | -0.611 | 0.349 |
| LOC102723407 | 102723407 | 3.788 | 0.351 |
| MMP9 | 4318 | 3.856 | 0.352 |
| DSG1 | 1828 | -3.793 | 0.356 |
| YWHAZ | 7534 | 4.012 | 0.358 |
| SERPINF1 | 5176 | 0.111 | 0.358 |
| APOA4 | 337 | -0.353 | 0.362 |
| VCL | 7414 | 3.294 | 0.362 |
| ALCAM | 214 | 4.037 | 0.368 |
| LAMB1 | 3912 | 3.504 | 0.368 |
| P4HB | 5034 | 3.182 | 0.370 |
| CFB | 629 | -0.205 | 0.373 |
| FGG | 2266 | 3.706 | 0.375 |
| EXTL2 | 2135 | -4.704 | 0.376 |
| SERPINA6 | 866 | -0.135 | 0.376 |
| RTEL1 | 51750 | 3.846 | 0.378 |
| GGH | 8836 | 0.567 | 0.381 |
| DPEP2 | 64174 | 4.097 | 0.382 |
| FCN3 | 8547 | -0.848 | 0.383 |
| FCN2 | 2220 | -0.417 | 0.383 |
| HLA-C | 3107 | -3.876 | 0.383 |
| SLC3A2 | 6520 | 2.706 | 0.384 |
| LDHA | 3939 | 4.031 | 0.386 |
| ITGB1 | 3688 | 3.325 | 0.387 |
| ISLR | 3671 | 3.715 | 0.387 |
| LAMP2 | 3920 | 4.465 | 0.390 |
| C4BPA | 722 | -0.397 | 0.392 |
| CRP | 1401 | -2.217 | 0.393 |
| OAF | 220323 | 4.252 | 0.394 |
| PAM | 5066 | 3.205 | 0.395 |
| KIT | 3815 | 2.991 | 0.395 |
| TGFB1 | 7040 | -3.572 | 0.397 |
| HBA1 | 3039 | 0.845 | 0.399 |
| HBA2 | 3040 | 0.845 | 0.399 |
| PFN1 | 5216 | 3.890 | 0.400 |
| TXN | 7295 | 4.529 | 0.400 |
| CTSF | 8722 | 3.808 | 0.403 |
| BASP1 | 10409 | 3.856 | 0.403 |
| C1QB | 713 | -1.118 | 0.405 |
| PCOLCE | 5118 | 0.964 | 0.406 |
| LTA4H | 4048 | -3.348 | 0.407 |
| EPHA4 | 2043 | 2.469 | 0.407 |
| PVR | 5817 | 4.310 | 0.410 |
| IGFBP7 | 3490 | 3.506 | 0.411 |
| TMSB4X | 7114 | -5.513 | 0.411 |
| TLN1 | 7094 | 0.383 | 0.412 |
| CLSTN1 | 22883 | 3.012 | 0.413 |
| FAM3C | 10447 | -2.802 | 0.413 |
| CPB2 | 1361 | 0.252 | 0.413 |
| ARHGAP35 | 2909 | 4.127 | 0.414 |
| PRG2 | 5553 | 3.820 | 0.414 |
| MMP2 | 4313 | -0.495 | 0.415 |
| F2 | 2147 | 0.155 | 0.416 |
| IHH | 3549 | 3.211 | 0.416 |
| CCDC126 | 90693 | 3.680 | 0.418 |
| UTS2 | 10911 | 4.517 | 0.418 |
| DKK3 | 27122 | 3.819 | 0.419 |
| STOM | 2040 | 0.027 | 0.423 |
| SH3BGRL3 | 83442 | -0.049 | 0.423 |
| CHI3L1 | 1116 | 0.096 | 0.423 |
| TAGLN2 | 8407 | 0.181 | 0.423 |
| ENO1 | 2023 | -0.258 | 0.423 |
| RAP1A | 5906 | -0.470 | 0.423 |
| PTPRK | 5796 | 0.289 | 0.423 |
| JUP | 3728 | -0.633 | 0.423 |
| HSPA8 | 3312 | 0.423 | 0.423 |
| VAT1 | 10493 | 0.287 | 0.423 |
| TUBB | 203068 | -1.017 | 0.423 |
| NEBL | 10529 | -1.161 | 0.423 |
| TUBB2A | 7280 | -1.017 | 0.423 |
| GALNT2 | 2590 | -2.349 | 0.423 |
| TUBB2B | 347733 | -1.017 | 0.423 |
| TUBB4A | 10382 | -1.017 | 0.423 |
| TUBB4B | 10383 | -1.017 | 0.423 |
| HIST1H1A | 3024 | -3.172 | 0.423 |
| TUBA8 | 51807 | -2.381 | 0.423 |
| TUBA1A | 7846 | -2.381 | 0.423 |
| MRC1 | 4360 | -2.091 | 0.423 |
| PSMA3 | 5684 | 3.054 | 0.423 |
| ENG | 2022 | -2.474 | 0.423 |
| DOCK7 | 85440 | -2.787 | 0.423 |
| ATP1A1 | 476 | -1.761 | 0.423 |
| ATP1A3 | 478 | -1.761 | 0.423 |
| MYH1 | 4619 | -2.627 | 0.423 |
| SUMO4 | 387082 | 3.004 | 0.423 |
| SUMO3 | 6612 | 3.004 | 0.423 |
| CLEC4M | 10332 | -2.099 | 0.423 |
| HIST4H4 | 121504 | -2.629 | 0.423 |
| HIST2H4B | 554313 | -2.629 | 0.423 |
| HIST1H4I | 8294 | -2.629 | 0.423 |
| HIST1H4A | 8359 | -2.629 | 0.423 |
| HIST1H4D | 8360 | -2.629 | 0.423 |
| HIST1H4F | 8361 | -2.629 | 0.423 |
| HIST1H4K | 8362 | -2.629 | 0.423 |
| HIST1H4J | 8363 | -2.629 | 0.423 |
| HIST1H4C | 8364 | -2.629 | 0.423 |
| HIST1H4H | 8365 | -2.629 | 0.423 |
| HIST1H4B | 8366 | -2.629 | 0.423 |
| HIST1H4E | 8367 | -2.629 | 0.423 |
| HIST1H4L | 8368 | -2.629 | 0.423 |
| HIST2H4A | 8370 | -2.629 | 0.423 |
| CAMP | 820 | -3.013 | 0.423 |
| LGALS3 | 3958 | 3.278 | 0.423 |
| TUBB1 | 81027 | -3.348 | 0.423 |
| ENPEP | 2028 | 2.709 | 0.423 |
| RAP1B | 5908 | -0.470 | 0.423 |
| TUBA4A | 7277 | -2.420 | 0.423 |
| AXL | 558 | -2.915 | 0.423 |
| TUBB8P12 | 260334 | -2.666 | 0.423 |
| LSG1 | 55341 | -2.909 | 0.423 |
| SEMA4B | 10509 | -3.015 | 0.423 |
| SDC4 | 6385 | 3.330 | 0.423 |
| NUP153 | 9972 | -4.637 | 0.423 |
| GOLM1 | 51280 | -2.993 | 0.423 |
| PCSK1N | 27344 | -3.693 | 0.423 |
| CFHR4 | 10877 | 5.083 | 0.423 |
| SUMO2 | 6613 | 3.111 | 0.423 |
| CTSB | 1508 | -2.584 | 0.423 |
| AK1 | 203 | 3.628 | 0.423 |
| OIT3 | 170392 | 2.963 | 0.423 |
| F8 | 2157 | 1.705 | 0.423 |
| ERP44 | 23071 | 2.757 | 0.423 |
| UBE2V2 | 7336 | 3.075 | 0.423 |
| ALAD | 210 | 3.630 | 0.423 |
| TMEM189-UBE2V1 | 387522 | 2.727 | 0.423 |
| SNCA | 6622 | 3.494 | 0.423 |
| IGF2R | 3482 | 2.516 | 0.423 |
| MYH2 | 4620 | -2.473 | 0.423 |
| LILRA3 | 11026 | -1.497 | 0.423 |
| PM20D1 | 148811 | -0.516 | 0.423 |
| CAP1 | 10487 | 3.288 | 0.423 |
| CTSZ | 1522 | 3.353 | 0.423 |
| HIST1H1T | 3010 | -3.065 | 0.423 |
| B3GNT8 | 374907 | 3.614 | 0.423 |
| PLXND1 | 23129 | 2.204 | 0.423 |
| KRT3 | 3850 | -4.990 | 0.423 |
| KRT19 | 3880 | -3.720 | 0.423 |
| ASXL3 | 80816 | -4.129 | 0.423 |
| FBLN5 | 10516 | 2.827 | 0.423 |
| TTC30B | 150737 | 3.265 | 0.423 |
| ATF6 | 22926 | 2.766 | 0.423 |
| RCN1 | 5954 | 2.756 | 0.423 |
| UGT8 | 7368 | -4.973 | 0.423 |
| ANTXR1 | 84168 | 2.879 | 0.423 |
| CD9 | 928 | 3.915 | 0.423 |
| CHMP4B | 128866 | -5.140 | 0.423 |
| ZNF550 | 162972 | 4.548 | 0.423 |
| ZG16B | 124220 | 2.894 | 0.423 |
| COL6A1 | 1291 | -4.039 | 0.423 |
| ADSL | 158 | 3.619 | 0.423 |
| KRT78 | 196374 | -3.480 | 0.423 |
| PRDX5 | 25824 | 3.066 | 0.423 |
| PARVB | 29780 | -3.421 | 0.423 |
| HIST1H1E | 3008 | -3.019 | 0.423 |
| PLEK | 5341 | 3.307 | 0.423 |
| PODXL | 5420 | 3.447 | 0.423 |
| PSMA6 | 5687 | -3.486 | 0.423 |
| HYI | 81888 | 6.686 | 0.423 |
| TUBA1C | 84790 | -2.339 | 0.423 |
| CR2 | 1380 | 0.249 | 0.423 |
| MYH9 | 4627 | -1.932 | 0.423 |
| OGN | 4969 | 3.372 | 0.423 |
| CA3 | 761 | -3.985 | 0.423 |
| ACTG2 | 72 | 4.668 | 0.423 |
| GGCT | 79017 | -3.767 | 0.423 |
| TIMP1 | 7076 | 4.090 | 0.423 |
| PRDX1 | 5052 | 4.057 | 0.423 |
| ARHGDIA | 396 | 3.760 | 0.423 |
| CALM2 | 805 | 3.754 | 0.423 |
| UBE2V1 | 7335 | 3.184 | 0.423 |
| PSMA7 | 5688 | 3.557 | 0.423 |
| TREH | 11181 | 3.710 | 0.423 |
| SPTB | 6710 | 2.253 | 0.423 |
| PEBP1 | 5037 | 3.204 | 0.423 |
| S100A4 | 6275 | 3.329 | 0.423 |
| NEO1 | 4756 | 0.701 | 0.423 |
| CTSG | 1511 | 3.002 | 0.423 |
| FAM50A | 9130 | -4.593 | 0.423 |
| TUBA1B | 10376 | -2.371 | 0.423 |
| TUBA3E | 112714 | -2.371 | 0.423 |
| TUBA3D | 113457 | -2.371 | 0.423 |
| HIST1H1C | 3006 | -2.977 | 0.423 |
| BNIP3 | 664 | 4.112 | 0.423 |
| TUBA3C | 7278 | -2.371 | 0.423 |
| BPNT1 | 10380 | -3.909 | 0.423 |
| CDSN | 1041 | 3.778 | 0.423 |
| KRT72 | 140807 | -4.136 | 0.423 |
| KRT80 | 144501 | 4.670 | 0.423 |
| H1F0 | 3005 | -3.297 | 0.423 |
| S100A12 | 6283 | -3.689 | 0.423 |
| CHMP4C | 92421 | -5.104 | 0.423 |
| ADAMTSL2 | 9719 | -3.296 | 0.423 |
| C1QTNF3 | 114899 | 3.061 | 0.423 |
| CNTFR | 1271 | -3.197 | 0.423 |
| CECR1 | 51816 | -3.636 | 0.423 |
| CD209 | 30835 | -2.140 | 0.423 |
| CKM | 1158 | 3.704 | 0.423 |
| COL18A1 | 80781 | 2.831 | 0.423 |
| ZNF292 | 23036 | -2.123 | 0.423 |
| TPM3 | 7170 | -3.920 | 0.423 |
| S100A7A | 338324 | -3.154 | 0.423 |
| S100A7 | 6278 | -3.154 | 0.423 |
| YWHAG | 7532 | -3.105 | 0.423 |
| ERBB4 | 2066 | 4.145 | 0.423 |
| ERBB2 | 2064 | 4.265 | 0.423 |
| ACTA2 | 59 | 4.607 | 0.423 |
| TUBB8 | 347688 | -2.634 | 0.423 |
| HIST1H1D | 3007 | -2.939 | 0.423 |
| WARS | 7453 | 3.281 | 0.423 |
| PSMA5 | 5686 | 3.557 | 0.423 |
| CALM3 | 808 | 3.816 | 0.423 |
| CALM1 | 801 | 3.771 | 0.423 |
| PNP | 4860 | 2.904 | 0.423 |
| SPTA1 | 6708 | 1.192 | 0.423 |
| MYH4 | 4622 | -2.129 | 0.423 |
| HLA-E | 3133 | -2.604 | 0.423 |
| PITPNM2 | 57605 | 2.353 | 0.423 |
| INPP5D | 3635 | -4.059 | 0.423 |
| KRT13 | 3860 | -3.595 | 0.423 |
| TUBB6 | 84617 | -2.675 | 0.423 |
| TKT | 7086 | 3.014 | 0.423 |
| HSP90AA1 | 3320 | 2.624 | 0.423 |
| LGALS7 | 3963 | 2.199 | 0.423 |
| PTPRF | 5792 | 2.046 | 0.423 |
| LGALS7B | 653499 | 2.199 | 0.423 |
| TXNDC15 | 79770 | 2.955 | 0.423 |
| LUZP1 | 7798 | 2.216 | 0.423 |
| ADGRE5 | 976 | 2.214 | 0.423 |
| CSPG4 | 1464 | -1.668 | 0.423 |
| ATP1A2 | 477 | -1.739 | 0.423 |
| CNTN3 | 5067 | -2.715 | 0.423 |
| CDHR5 | 53841 | 2.618 | 0.423 |
| HPRT1 | 3251 | 0.177 | 0.423 |
| LMAN1 | 3998 | 2.079 | 0.423 |
| NUCB1 | 4924 | -2.344 | 0.423 |
| CPS1 | 1373 | -2.441 | 0.423 |
| AOC2 | 314 | 1.890 | 0.423 |
| MARCO | 8685 | 0.824 | 0.423 |
| KDM3A | 55818 | -0.271 | 0.423 |
| PGK1 | 5230 | 0.340 | 0.423 |
| FERMT3 | 83706 | -0.237 | 0.423 |
| C4A | 720 | 0.155 | 0.423 |
| FLNA | 2316 | 3.193 | 0.423 |
| CTSC | 1075 | -3.187 | 0.423 |
| COLEC10 | 10584 | -0.480 | 0.425 |
| CA2 | 760 | 1.169 | 0.427 |
| BLVRB | 645 | 1.591 | 0.429 |
| SERPING1 | 710 | -0.090 | 0.430 |
| GSTO1 | 9446 | 4.155 | 0.431 |
| ABI3BP | 25890 | 2.637 | 0.432 |
| HBB | 3043 | 0.755 | 0.435 |
| PLA2G7 | 7941 | 3.619 | 0.439 |
| TGOLN2 | 10618 | -2.960 | 0.439 |
| S100A8 | 6279 | -0.349 | 0.441 |
| PPIA | 5478 | -4.034 | 0.443 |
| SELP | 6403 | -2.750 | 0.443 |
| ITGA2B | 3674 | 2.991 | 0.445 |
| SFTPA1 | 653509 | 2.910 | 0.445 |
| SFTPA2 | 729238 | 2.910 | 0.445 |
| HYAL1 | 3373 | 3.814 | 0.449 |
| KRT10 | 3858 | -0.882 | 0.449 |
| DAG1 | 1605 | 3.610 | 0.450 |
| PEBP4 | 157310 | -2.983 | 0.451 |
| BCHE | 590 | 0.222 | 0.452 |
| DCD | 117159 | 1.008 | 0.452 |
| SEPP1 | 6414 | 0.142 | 0.454 |
| APCS | 325 | -0.290 | 0.455 |
| ANPEP | 290 | 0.547 | 0.456 |
| ACTA1 | 58 | -0.561 | 0.458 |
| ACTC1 | 70 | -0.561 | 0.458 |
| SERPINA4 | 5267 | 0.242 | 0.459 |
| WDR1 | 9948 | 2.410 | 0.459 |
| CTSH | 1512 | 2.697 | 0.460 |
| TF | 7018 | -0.174 | 0.463 |
| CD5L | 922 | -3.756 | 0.465 |
| ATRN | 8455 | 0.414 | 0.465 |
| PCSK9 | 255738 | 1.054 | 0.469 |
| CD14 | 929 | 0.254 | 0.470 |
| APOH | 350 | -0.157 | 0.470 |
| APOA1 | 335 | 0.164 | 0.473 |
| PPBP | 5473 | -0.508 | 0.474 |
| FRMPD1 | 22844 | 3.447 | 0.476 |
| FUCA1 | 2517 | 0.629 | 0.477 |
| RNASE4 | 6038 | -3.922 | 0.477 |
| ERAP2 | 64167 | 2.363 | 0.478 |
| FUCA2 | 2519 | 2.064 | 0.478 |
| HGFAC | 3083 | 0.273 | 0.478 |
| MRC2 | 9902 | 2.200 | 0.480 |
| GAPDH | 2597 | 3.956 | 0.482 |
| LPA | 4018 | -0.104 | 0.482 |
| COLEC11 | 78989 | 3.892 | 0.484 |
| LCP1 | 3936 | -0.163 | 0.484 |
| KRT6A | 3853 | -4.889 | 0.485 |
| FMN2 | 56776 | 3.033 | 0.486 |
| MPO | 4353 | 2.791 | 0.489 |
| ORM1 | 5004 | 0.619 | 0.490 |
| S100A6 | 6277 | 0.507 | 0.490 |
| PZP | 5858 | -0.403 | 0.491 |
| CD163 | 9332 | 2.856 | 0.495 |
| OLFM1 | 10439 | -3.124 | 0.497 |
| CALR | 811 | 3.360 | 0.497 |
| PF4 | 5196 | -0.460 | 0.501 |
| PF4V1 | 5197 | -0.460 | 0.501 |
| CD109 | 135228 | -2.266 | 0.502 |
| FGA | 2243 | -0.589 | 0.504 |
| LTF | 4057 | -2.431 | 0.507 |
| LMAN2 | 10960 | -2.772 | 0.507 |
| C1QC | 714 | -0.406 | 0.507 |
| SBSN | 374897 | 2.114 | 0.507 |
| C8A | 731 | 0.344 | 0.509 |
| CLU | 1191 | 0.067 | 0.515 |
| CD44 | 960 | 0.372 | 0.515 |
| C1RL | 51279 | 0.402 | 0.515 |
| F13A1 | 2162 | -3.512 | 0.517 |
| ITIH2 | 3698 | -0.155 | 0.518 |
| ALDOB | 229 | 1.235 | 0.518 |
| KRT5 | 3852 | -0.851 | 0.518 |
| ADIPOQ | 9370 | 0.187 | 0.519 |
| CA1 | 759 | 0.728 | 0.519 |
| INHBC | 3626 | 0.353 | 0.522 |
| C8G | 733 | -0.375 | 0.524 |
| PEPD | 5184 | 3.443 | 0.529 |
| CAT | 847 | 1.041 | 0.531 |
| CALU | 813 | -3.561 | 0.532 |
| CES1 | 1066 | 1.951 | 0.532 |
| APOE | 348 | -0.163 | 0.533 |
| RPS27A | 6233 | -2.908 | 0.537 |
| C5 | 727 | -0.379 | 0.539 |
| UBA52 | 7311 | -2.834 | 0.540 |
| PROZ | 8858 | 0.192 | 0.545 |
| PGLYRP2 | 114770 | 0.153 | 0.549 |
| UBB | 7314 | -2.501 | 0.552 |
| B3GNT2 | 10678 | -2.855 | 0.558 |
| CFHR5 | 81494 | 0.133 | 0.559 |
| MASP2 | 10747 | -0.217 | 0.562 |
| APOD | 347 | 0.259 | 0.565 |
| F10 | 2159 | 0.139 | 0.567 |
| RBP4 | 5950 | 0.059 | 0.568 |
| CHGA | 1113 | 2.272 | 0.569 |
| C4BPB | 725 | -0.223 | 0.570 |
| APOF | 319 | -0.216 | 0.572 |
| APMAP | 57136 | 0.441 | 0.572 |
| SERPINA3 | 12 | -0.154 | 0.572 |
| ALDOA | 226 | -1.883 | 0.575 |
| ORM2 | 5005 | 0.409 | 0.575 |
| ZFP14 | 57677 | -0.531 | 0.580 |
| UBC | 7316 | -1.950 | 0.580 |
| SERPINC1 | 462 | 0.145 | 0.585 |
| C4B | 721 | -0.220 | 0.587 |
| ANG | 283 | -2.445 | 0.590 |
| A1BG | 1 | 0.062 | 0.596 |
| LBP | 3929 | -0.391 | 0.599 |
| MASP1 | 5648 | 0.119 | 0.600 |
| SERPINA7 | 6906 | 0.100 | 0.602 |
| CST3 | 1471 | -0.102 | 0.615 |
| CPN2 | 1370 | 0.153 | 0.618 |
| SERPINA1 | 5265 | 0.086 | 0.618 |
| ANGPTL3 | 27329 | 0.525 | 0.620 |
| CSF1R | 1436 | 3.172 | 0.622 |
| IGFBP3 | 3486 | 0.133 | 0.624 |
| COL6A3 | 1293 | 0.224 | 0.625 |
| IGFBP5 | 3488 | 4.457 | 0.626 |
| BTD | 686 | 0.149 | 0.626 |
| HLA-A | 3105 | 2.415 | 0.632 |
| VTN | 7448 | -0.183 | 0.635 |
| GPI | 2821 | 1.337 | 0.642 |
| ITIH3 | 3699 | 0.110 | 0.643 |
| IL1RAP | 3556 | 0.700 | 0.644 |
| SVEP1 | 79987 | -1.145 | 0.650 |
| HRNR | 388697 | 0.224 | 0.650 |
| PON1 | 5444 | -0.063 | 0.653 |
| LECT2 | 3950 | 4.193 | 0.658 |
| ENDOD1 | 23052 | 2.416 | 0.661 |
| KRT1 | 3848 | -0.391 | 0.662 |
| LCAT | 3931 | 0.218 | 0.667 |
| GC | 2638 | 0.141 | 0.669 |
| GP5 | 2814 | 1.106 | 0.671 |
| GNPTG | 84572 | 0.335 | 0.678 |
| ECM1 | 1893 | -0.249 | 0.681 |
| ADAMDEC1 | 27299 | -2.865 | 0.685 |
| MAN1A1 | 4121 | 0.219 | 0.691 |
| NCAM1 | 4684 | -0.071 | 0.691 |
| KRT77 | 374454 | -3.227 | 0.692 |
| FLT4 | 2324 | 0.306 | 0.693 |
| QSOX1 | 5768 | 0.128 | 0.694 |
| APOL1 | 8542 | -0.109 | 0.697 |
| FCGBP | 8857 | -0.161 | 0.706 |
| HSPA6 | 3310 | 0.178 | 0.707 |
| DSG2 | 1829 | 0.572 | 0.709 |
| CASP14 | 23581 | 3.046 | 0.711 |
| GM2A | 2760 | 2.725 | 0.713 |
| LRG1 | 116844 | 0.159 | 0.715 |
| KRT33A | 3883 | 2.693 | 0.723 |
| CETP | 1071 | -3.507 | 0.724 |
| LOC100653049 | 100653049 | 2.644 | 0.724 |
| KRT34 | 3885 | 2.644 | 0.724 |
| F7 | 2155 | 0.185 | 0.725 |
| F9 | 2158 | 0.145 | 0.730 |
| C8B | 732 | -0.207 | 0.734 |
| HSPG2 | 3339 | 0.439 | 0.735 |
| LTBP1 | 4052 | -0.621 | 0.738 |
| PDE7A | 5150 | -0.066 | 0.745 |
| FBLN1 | 2192 | -0.137 | 0.745 |
| KRT17 | 3872 | -1.641 | 0.745 |
| CDH5 | 1003 | 0.075 | 0.749 |
| AMBP | 259 | -0.047 | 0.750 |
| AGT | 183 | -0.109 | 0.756 |
| SRGN | 5552 | -0.549 | 0.761 |
| C1QA | 712 | 0.270 | 0.762 |
| KRT9 | 3857 | -0.149 | 0.768 |
| HP | 3240 | 0.123 | 0.775 |
| LCN2 | 3934 | -0.052 | 0.779 |
| PLTP | 5360 | -0.176 | 0.780 |
| AFM | 173 | 0.083 | 0.781 |
| GSN | 2934 | -0.050 | 0.790 |
| HYOU1 | 10525 | -0.110 | 0.790 |
| GPX3 | 2878 | 0.181 | 0.790 |
| TFRC | 7037 | -0.053 | 0.793 |
| SAA2-SAA4 | 100528017 | 0.047 | 0.796 |
| TGFBI | 7045 | 0.207 | 0.798 |
| MINPP1 | 9562 | -0.139 | 0.800 |
| MMRN2 | 79812 | 0.160 | 0.806 |
| APOC1 | 341 | 0.214 | 0.811 |
| AOC3 | 8639 | -1.205 | 0.812 |
| FLG2 | 388698 | -1.188 | 0.816 |
| CPN1 | 1369 | -0.112 | 0.827 |
| ALB | 213 | 0.032 | 0.829 |
| SERPINA5 | 5104 | 0.121 | 0.832 |
| A2M | 2 | -0.101 | 0.839 |
| POSTN | 10631 | -0.051 | 0.842 |
| CLEC3B | 7123 | -0.042 | 0.843 |
| C1R | 715 | 0.097 | 0.845 |
| NID1 | 4811 | 1.135 | 0.847 |
| TNC | 3371 | 0.167 | 0.849 |
| EFEMP1 | 2202 | 0.375 | 0.850 |
| VCAM1 | 7412 | -0.099 | 0.857 |
| SERPINA10 | 51156 | 0.066 | 0.859 |
| NRP1 | 8829 | -0.207 | 0.860 |
| HLA-B | 3106 | 1.115 | 0.863 |
| HPX | 3263 | -0.028 | 0.864 |
| ICAM1 | 3383 | 0.115 | 0.868 |
| LDHB | 3945 | 0.087 | 0.870 |
| PLXDC2 | 84898 | 0.073 | 0.871 |
| PTPRG | 5793 | -0.066 | 0.871 |
| PROC | 5624 | 0.082 | 0.872 |
| YWHAE | 7531 | 0.806 | 0.879 |
| SERPINF2 | 5345 | -0.011 | 0.886 |
| FCGR3A | 2214 | -0.283 | 0.899 |
| FLG | 2312 | 0.499 | 0.901 |
| APOA2 | 336 | 0.047 | 0.903 |
| MST1 | 4485 | 0.057 | 0.906 |
| HRG | 3273 | 0.032 | 0.906 |
| IGFALS | 3483 | -0.030 | 0.909 |
| VWF | 7450 | -0.045 | 0.911 |
| C2 | 717 | -0.018 | 0.912 |
| IL6ST | 3572 | 0.043 | 0.913 |
| COMP | 1311 | 0.084 | 0.913 |
| FETUB | 26998 | 0.050 | 0.914 |
| ITIH4 | 3700 | 0.022 | 0.916 |
| ACTB | 60 | -0.041 | 0.918 |
| ACTG1 | 71 | -0.041 | 0.918 |
| GP1BA | 2811 | 0.089 | 0.918 |
| H6PD | 9563 | -0.640 | 0.919 |
| APP | 351 | 0.164 | 0.919 |
| AMY1A | 276 | 0.323 | 0.921 |
| AMY1B | 277 | 0.323 | 0.921 |
| AMY1C | 278 | 0.323 | 0.921 |
| AMY2A | 279 | 0.323 | 0.921 |
| AMY2B | 280 | 0.323 | 0.921 |
| THBS4 | 7060 | -0.053 | 0.923 |
| CNDP1 | 84735 | 0.013 | 0.924 |
| PON3 | 5446 | 0.022 | 0.926 |
| APOM | 55937 | -0.030 | 0.927 |
| HLA-G | 3135 | -0.012 | 0.929 |
| FN1 | 2335 | -0.039 | 0.931 |
| MAN2A1 | 4124 | -0.493 | 0.933 |
| SERPIND1 | 3053 | -0.021 | 0.939 |
| CDH13 | 1012 | -0.013 | 0.942 |
| C9 | 735 | -0.024 | 0.945 |
| UTY | 7404 | 0.460 | 0.945 |
| KDM6A | 7403 | 0.460 | 0.945 |
| ACTN1 | 87 | -0.356 | 0.950 |
| FAH | 2184 | 0.437 | 0.951 |
| KRT6C | 286887 | -0.654 | 0.952 |
| PTGDS | 5730 | 0.005 | 0.959 |
| LUM | 4060 | -0.011 | 0.962 |
| QPCT | 25797 | -0.334 | 0.962 |
| CADM1 | 23705 | -0.235 | 0.970 |
| PDIA3 | 2923 | 0.193 | 0.973 |
| LAMA2 | 3908 | 0.100 | 0.974 |
| FCGR3B | 2215 | -0.232 | 0.974 |
| LAMP1 | 3916 | 0.214 | 0.975 |
| ACTBL2 | 345651 | 0.289 | 0.977 |
| CD99 | 4267 | -0.133 | 0.985 |
| PTPRB | 5787 | -0.068 | 0.985 |
| VNN1 | 8876 | 0.007 | 0.986 |
| PRCP | 5547 | -0.112 | 0.986 |
| ADAMTS13 | 11093 | 0.018 | 0.987 |
| TIMP2 | 7077 | -0.125 | 0.987 |
| HABP2 | 3026 | -0.005 | 0.987 |
| FGFR1 | 2260 | -0.063 | 0.988 |
| F12 | 2161 | -0.005 | 0.989 |
| CFHR3 | 10878 | -0.061 | 0.992 |
| CDH1 | 999 | -0.049 | 0.993 |
| ENPP2 | 5168 | -0.002 | 0.997 |
| CFD | 1675 | 0.000 | 0.998 |
| UNC5B | 219699 | 0.004 | 1.000 |
