# Supplementary material for: Decreased proliferation of HepG2 liver cancer cells in vitro and exhibited proteomic changes in vivo in subjects with metabolic syndrome and metabolic dysfunction-associated steatotic liver disease who performed four-week dawn-to-dusk dry fasting
Source: Clin Proteomics. 2025 Jun 24;22:25. doi: 10.1186/s12014-025-09547-3 (PMC12186377; doi:10.1186/s12014-025-09547-3)
Supplement: Supplementary file 3 — Additional file 3 [file 12014_2025_9547_MOESM3_ESM.docx]

**Supplemental Table 3.** Peptide Spectrum Match (PSM) of Differentially Expressed Gene Protein Products (GP) in Serum Collected from Healthy Subjects Who Performed 4-Week Dawn-to-Dusk Dry Fasting (DDDF)

|  |  | **Before 4-Week DDDF** | | | | **At the End of 4-Week DDDF** | | | |
| --- | --- | --- | --- | --- | --- | --- | --- | --- | --- |
| **Gene ID** | **Gene Symbol** | **Subject 1** | **Subject 2** | **Subject 3** | **Subject 4** | **Subject 1** | **Subject 2** | **Subject 3** | **Subject 4** |
| 29082 | CHMP4A | 6 | 10 | 7 | 6 | 8 | 9 | 8 | 10 |
| 23105 | FSTL4 | 1 | 1 | 1 | 1 | ND | ND | 3 | ND |
| 1475 | CSTA | 2 | 1 | 2 | 4 | ND | ND | 2 | ND |
| 1075 | CTSC | 2 | 1 | ND | 2 | ND | ND | ND | ND |
| 3934 | LCN2 | 2 | 1 | 5 | 2 | ND | 2 | 5 | ND |
| 2171 | FABP5 | 2 | 1 | 1 | 3 | ND | ND | ND | ND |
| 5216 | PFN1 | 4 | 7 | 8 | 2 | ND | ND | ND | ND |
| 7531 | YWHAE | 5 | 6 | 7 | 1 | ND | ND | 1 | 2 |
| 135228 | CD109 | 6 | 3 | 1 | 6 | 6 | 1 | 4 | 3 |
| 22918 | CD93 | 5 | 5 | 2 | 6 | 3 | 2 | 5 | 4 |
| 2316 | FLNA | 46 | 46 | 32 | 1 | ND | ND | ND | ND |
