# Supplementary material for: Decreased proliferation of HepG2 liver cancer cells in vitro and exhibited proteomic changes in vivo in subjects with metabolic syndrome and metabolic dysfunction-associated steatotic liver disease who performed four-week dawn-to-dusk dry fasting
Source: Clin Proteomics. 2025 Jun 24;22:25. doi: 10.1186/s12014-025-09547-3 (PMC12186377; doi:10.1186/s12014-025-09547-3)
Supplement: Supplementary file 4 — Additional file 4 [file 12014_2025_9547_MOESM4_ESM.docx]

| **Supplementary Table S4**. Serum Gene Protein Product (GP) Levels that Increased or Decreased at the End of 4-Week Dawn-to-Dusk Dry Fasting (V2) Compared with the GP Levels Before 4-Week Dawn-to-Dusk Dry Fasting (V1) in Healthy Subjects | | | |
| --- | --- | --- | --- |
| **Gene Symbol** | **Gene ID** | **Average Paired Log2 Fold Change (V2/V1)** | **Paired P Value** |
| CHMP4A | 29082 | 0.661 | 0.045 |
| FLNA | 2316 | -10.331 | 0.0498 |
| CD93 | 22918 | -2.567 | 0.041 |
| CD109 | 135228 | -1.321 | 0.039 |
| YWHAE | 7531 | -9.769 | 0.026 |
| PFN1 | 5216 | -12.865 | 0.012 |
| FABP5 | 2171 | -12.127 | 0.010 |
| LCN2 | 3934 | -11.271 | 0.002 |
| CTSC | 1075 | -8.542 | 0.001 |
| CSTA | 1475 | -12.325 | 0.0003 |
| FSTL4 | 23105 | -9.016 | 0.0002 |
| HSP90AA1 | 3320 | 0.001 | 1.000 |
| ISLR | 3671 | -0.010 | 0.999 |
| SEMA4B | 10509 | 0.017 | 0.998 |
| OMD | 4958 | -0.021 | 0.998 |
| L1CAM | 3897 | -0.037 | 0.993 |
| H6PD | 9563 | 0.067 | 0.992 |
| KRT2 | 3849 | -0.017 | 0.987 |
| HLA-G | 3135 | -0.131 | 0.987 |
| ADGRF5 | 221395 | 0.099 | 0.985 |
| C4B_2 | 100293534 | 0.002 | 0.983 |
| C4B | 721 | 0.002 | 0.983 |
| LAMP1 | 3916 | 0.161 | 0.981 |
| IGFBP5 | 3488 | 0.203 | 0.980 |
| FRMPD1 | 22844 | -0.170 | 0.980 |
| F2 | 2147 | 0.001 | 0.980 |
| GSN | 2934 | 0.004 | 0.979 |
| MPO | 4353 | -0.138 | 0.978 |
| CCDC126 | 90693 | -0.012 | 0.974 |
| INHBC | 3626 | -0.010 | 0.967 |
| TKT | 7086 | -0.239 | 0.967 |
| CTSD | 1509 | -0.327 | 0.964 |
| CFP | 5199 | 0.028 | 0.961 |
| MST1 | 4485 | 0.026 | 0.959 |
| FTH1 | 2495 | -0.264 | 0.959 |
| APOC4 | 346 | -0.041 | 0.951 |
| GP1BA | 2811 | -0.021 | 0.950 |
| KDM3A | 55818 | -0.494 | 0.945 |
| LTA4H | 4048 | -0.356 | 0.944 |
| CD248 | 57124 | -0.454 | 0.931 |
| UTS2 | 10911 | -0.048 | 0.925 |
| ENO1 | 2023 | -0.675 | 0.925 |
| CETP | 1071 | 0.080 | 0.925 |
| FMN2 | 56776 | 0.587 | 0.923 |
| SOD3 | 6649 | 0.185 | 0.917 |
| ECM2 | 1842 | -0.467 | 0.916 |
| CFHR1 | 3078 | -0.043 | 0.916 |
| APOC2 | 344 | 0.029 | 0.916 |
| IGFBP6 | 3489 | 0.023 | 0.913 |
| GPI | 2821 | -0.731 | 0.910 |
| SERPINC1 | 462 | -0.020 | 0.907 |
| PCSK9 | 255738 | 0.086 | 0.898 |
| APOL1 | 8542 | -0.036 | 0.878 |
| APMAP | 57136 | -0.119 | 0.872 |
| FCN3 | 8547 | -0.055 | 0.872 |
| A2M | 2 | 0.072 | 0.859 |
| C6 | 729 | -0.035 | 0.858 |
| BASP1 | 10409 | 1.222 | 0.851 |
| ENPEP | 2028 | -0.976 | 0.848 |
| KNG1 | 3827 | 0.051 | 0.838 |
| VCAM1 | 7412 | 0.039 | 0.821 |
| ICAM1 | 3383 | 0.146 | 0.817 |
| QSOX1 | 5768 | -0.057 | 0.812 |
| LOC110384692 | 110384692 | 0.021 | 0.805 |
| APCS | 325 | 0.031 | 0.804 |
| ALB | 213 | -0.075 | 0.804 |
| F13B | 2165 | 0.070 | 0.802 |
| RBP4 | 5950 | -0.113 | 0.802 |
| KRT17 | 3872 | -2.152 | 0.798 |
| APOC3 | 345 | 0.099 | 0.796 |
| PON3 | 5446 | -0.144 | 0.794 |
| SERPINF1 | 5176 | 0.014 | 0.793 |
| LCAT | 3931 | -0.118 | 0.793 |
| CLU | 1191 | 0.046 | 0.792 |
| CRISP3 | 10321 | -0.411 | 0.783 |
| NEO1 | 4756 | -0.479 | 0.779 |
| APOM | 55937 | -0.183 | 0.777 |
| C1S | 716 | 0.013 | 0.774 |
| LGALS3BP | 3959 | -0.118 | 0.759 |
| HPX | 3263 | -0.083 | 0.754 |
| AZGP1 | 563 | -0.089 | 0.749 |
| FGFR1 | 2260 | -2.270 | 0.745 |
| SNED1 | 25992 | -1.604 | 0.744 |
| FBLN1 | 2192 | 0.073 | 0.740 |
| PI16 | 221476 | -0.059 | 0.738 |
| THBS4 | 7060 | 0.422 | 0.736 |
| MGP | 4256 | 0.572 | 0.732 |
| WARS | 7453 | -2.543 | 0.732 |
| F9 | 2158 | -0.131 | 0.728 |
| TF | 7018 | -0.042 | 0.725 |
| HP | 3240 | -0.069 | 0.715 |
| CP | 1356 | -0.057 | 0.711 |
| MASP1 | 5648 | -0.067 | 0.707 |
| ALDOB | 229 | -0.691 | 0.705 |
| ADIPOQ | 9370 | 0.217 | 0.703 |
| SAA1 | 6288 | -0.718 | 0.702 |
| P4HB | 5034 | -0.237 | 0.701 |
| HEG1 | 57493 | -0.254 | 0.696 |
| SERPINF2 | 5345 | 0.196 | 0.694 |
| LCP1 | 3936 | -0.274 | 0.694 |
| C5 | 727 | -0.083 | 0.691 |
| LILRA3 | 11026 | -2.505 | 0.681 |
| MBL2 | 4153 | -0.097 | 0.677 |
| PGLYRP1 | 8993 | -2.925 | 0.676 |
| LYZ | 4069 | 0.107 | 0.671 |
| PRCP | 5547 | -3.173 | 0.669 |
| DPP4 | 1803 | -0.499 | 0.667 |
| KRT10 | 3858 | -0.406 | 0.666 |
| MEGF8 | 1954 | -2.290 | 0.664 |
| NID1 | 4811 | -3.459 | 0.664 |
| CES1 | 1066 | -1.427 | 0.658 |
| PTPRB | 5787 | -2.144 | 0.656 |
| SHBG | 6462 | -0.186 | 0.654 |
| AHSG | 197 | 0.116 | 0.654 |
| HRG | 3273 | -0.099 | 0.652 |
| ANGPTL3 | 27329 | -0.391 | 0.642 |
| PGLYRP2 | 114770 | -0.108 | 0.636 |
| ITIH4 | 3700 | -0.061 | 0.635 |
| LUM | 4060 | -0.067 | 0.632 |
| PLG | 5340 | 0.087 | 0.631 |
| COL6A3 | 1293 | -0.136 | 0.629 |
| C8B | 732 | 0.087 | 0.623 |
| OLFM1 | 10439 | -0.262 | 0.622 |
| ITIH1 | 3697 | -0.106 | 0.614 |
| SPP2 | 6694 | -0.207 | 0.610 |
| PON1 | 5444 | -0.107 | 0.608 |
| ADAMTSL4 | 54507 | -0.775 | 0.605 |
| PDIA3 | 2923 | -0.322 | 0.605 |
| CTBS | 1486 | 0.180 | 0.604 |
| FGA | 2243 | -0.213 | 0.599 |
| CNTN1 | 1272 | -0.526 | 0.595 |
| C4BPA | 722 | -0.175 | 0.581 |
| C1R | 715 | -0.196 | 0.580 |
| ENPP2 | 5168 | 2.403 | 0.580 |
| ANG | 283 | 3.431 | 0.574 |
| SNCA | 6622 | -4.505 | 0.573 |
| PLTP | 5360 | 0.264 | 0.567 |
| NAGLU | 4669 | -2.798 | 0.565 |
| CFL1 | 1072 | -6.079 | 0.559 |
| FAM20C | 56975 | -2.258 | 0.554 |
| HYOU1 | 10525 | -0.239 | 0.554 |
| C3 | 718 | 0.033 | 0.545 |
| NCAM1 | 4684 | 0.439 | 0.543 |
| IGFBP7 | 3490 | -4.704 | 0.537 |
| GPLD1 | 2822 | 0.285 | 0.535 |
| PLA2G7 | 7941 | -3.210 | 0.532 |
| CORO1A | 11151 | -0.757 | 0.527 |
| ASGR2 | 433 | -3.352 | 0.525 |
| KRT16 | 3868 | -6.951 | 0.525 |
| C9 | 735 | -0.093 | 0.522 |
| HSP90B1 | 7184 | -0.480 | 0.520 |
| VASN | 114990 | 0.717 | 0.517 |
| SERPINA7 | 6906 | -0.077 | 0.511 |
| LTF | 4057 | -2.872 | 0.507 |
| POSTN | 10631 | 0.208 | 0.504 |
| SERPINA11 | 256394 | -4.082 | 0.503 |
| CNDP1 | 84735 | -0.198 | 0.500 |
| C1orf56 | 54964 | -0.148 | 0.498 |
| FGB | 2244 | 2.669 | 0.497 |
| LPA | 4018 | 0.159 | 0.497 |
| CFI | 3426 | 0.138 | 0.496 |
| IL6ST | 3572 | -2.621 | 0.494 |
| PROS1 | 5627 | 0.075 | 0.493 |
| ACE | 1636 | -0.175 | 0.492 |
| PRG2 | 5553 | -3.772 | 0.490 |
| KRT6B | 3854 | -4.790 | 0.490 |
| IGF2R | 3482 | -2.250 | 0.488 |
| COMP | 1311 | 0.595 | 0.485 |
| ADAMTS13 | 11093 | -0.450 | 0.484 |
| IGLL5 | 100423062 | -0.574 | 0.484 |
| CFH | 3075 | 0.131 | 0.483 |
| CD99 | 4267 | 3.413 | 0.482 |
| FSTL1 | 11167 | 3.169 | 0.482 |
| APOA5 | 116519 | -2.382 | 0.482 |
| AMY1A | 276 | -1.730 | 0.480 |
| AMY1B | 277 | -1.730 | 0.480 |
| AMY1C | 278 | -1.730 | 0.480 |
| TFRC | 7037 | -0.255 | 0.479 |
| EFEMP1 | 2202 | 0.284 | 0.475 |
| HPR | 3250 | -0.136 | 0.474 |
| EXTL2 | 2135 | 3.437 | 0.474 |
| B2M | 567 | 0.423 | 0.473 |
| FGG | 2266 | 2.429 | 0.472 |
| SOD2 | 6648 | -3.852 | 0.470 |
| LDHA | 3939 | -1.040 | 0.468 |
| B3GNT2 | 10678 | 2.968 | 0.462 |
| SERPIND1 | 3053 | -0.125 | 0.461 |
| ARHGDIB | 397 | -3.265 | 0.459 |
| ADGRL4 | 64123 | 2.902 | 0.457 |
| HSPG2 | 3339 | -1.664 | 0.456 |
| PEBP4 | 157310 | 3.558 | 0.456 |
| TTR | 7276 | -0.207 | 0.452 |
| SLC38A10 | 124565 | -1.892 | 0.450 |
| SAA2-SAA4 | 100528017 | 0.180 | 0.449 |
| HLA-C | 3107 | -3.716 | 0.448 |
| ALCAM | 214 | -3.494 | 0.448 |
| SERPINA3 | 12 | 0.052 | 0.445 |
| OGN | 4969 | 3.718 | 0.442 |
| VNN1 | 8876 | -3.637 | 0.442 |
| IGFBP2 | 3485 | -0.842 | 0.440 |
| VTN | 7448 | 0.143 | 0.439 |
| HABP2 | 3026 | 0.265 | 0.438 |
| HGFAC | 3083 | 0.157 | 0.436 |
| ENG | 2022 | -2.836 | 0.436 |
| DAG1 | 1605 | -1.021 | 0.432 |
| PTGDS | 5730 | -0.257 | 0.428 |
| LOC102723407 | 102723407 | -3.860 | 0.428 |
| GM2A | 2760 | 3.756 | 0.427 |
| PTPRG | 5793 | -0.433 | 0.425 |
| NRCAM | 4897 | -2.409 | 0.425 |
| FCGR3A | 2214 | -0.536 | 0.424 |
| PCYOX1 | 51449 | 0.228 | 0.424 |
| HLA-B | 3106 | -3.277 | 0.423 |
| CDH6 | 1004 | -3.247 | 0.423 |
| QPCT | 25797 | -0.015 | 0.423 |
| CAMP | 820 | -0.059 | 0.423 |
| KRT77 | 374454 | -0.106 | 0.423 |
| CNTN3 | 5067 | -0.112 | 0.423 |
| ST6GAL1 | 6480 | -0.143 | 0.423 |
| PDE7A | 5150 | 0.133 | 0.423 |
| KRT6C | 286887 | 0.251 | 0.423 |
| OAF | 220323 | -0.157 | 0.423 |
| ST3GAL6 | 10402 | 0.212 | 0.423 |
| XPNPEP2 | 7512 | 0.319 | 0.423 |
| F8 | 2157 | 0.385 | 0.423 |
| AMY2A | 279 | 0.184 | 0.423 |
| AMY2B | 280 | 0.184 | 0.423 |
| RTEL1 | 51750 | 0.529 | 0.423 |
| SCGB1A1 | 7356 | 0.219 | 0.423 |
| CHIT1 | 1118 | 1.616 | 0.423 |
| CASP14 | 23581 | -0.434 | 0.423 |
| PIANP | 196500 | 2.284 | 0.423 |
| ITGAL | 3683 | -1.302 | 0.423 |
| TUBA1A | 7846 | -2.615 | 0.423 |
| ADGRE5 | 976 | -2.376 | 0.423 |
| ITGA8 | 8516 | -2.645 | 0.423 |
| ZNF292 | 23036 | 1.725 | 0.423 |
| SHH | 6469 | 2.455 | 0.423 |
| GPX1 | 2876 | -2.830 | 0.423 |
| SUMO2 | 6613 | -3.272 | 0.423 |
| SUMO4 | 387082 | -3.165 | 0.423 |
| SUMO3 | 6612 | -3.165 | 0.423 |
| VAT1 | 10493 | -3.062 | 0.423 |
| ERBB4 | 2066 | 3.877 | 0.423 |
| PODXL | 5420 | -3.779 | 0.423 |
| VCAN | 1462 | 1.987 | 0.423 |
| ART3 | 419 | -3.472 | 0.423 |
| DSC2 | 1824 | 2.267 | 0.423 |
| PLXND1 | 23129 | -2.442 | 0.423 |
| TTN | 7273 | -1.828 | 0.423 |
| CKM | 1158 | 2.890 | 0.423 |
| ITGA2 | 3673 | 2.408 | 0.423 |
| KDR | 3791 | 1.995 | 0.423 |
| ART4 | 420 | 2.954 | 0.423 |
| EZR | 7430 | -2.849 | 0.423 |
| LGALS3 | 3958 | 3.277 | 0.423 |
| EIF5A2 | 56648 | -2.796 | 0.423 |
| LILRA1 | 11024 | -2.838 | 0.423 |
| TUBA1C | 84790 | -2.573 | 0.423 |
| EXT2 | 2132 | -2.680 | 0.423 |
| PCDH12 | 51294 | 2.809 | 0.423 |
| FGL2 | 10875 | 2.773 | 0.423 |
| ANGPTL6 | 83854 | 2.976 | 0.423 |
| LMAN2 | 10960 | 3.233 | 0.423 |
| HPD | 3242 | 3.285 | 0.423 |
| HAGH | 3029 | -3.010 | 0.423 |
| RANBP1 | 5902 | -3.612 | 0.423 |
| FBLN5 | 10516 | -3.104 | 0.423 |
| STOM | 2040 | -4.213 | 0.423 |
| COTL1 | 23406 | -4.094 | 0.423 |
| GP6 | 51206 | -3.486 | 0.423 |
| CA3 | 761 | -3.847 | 0.423 |
| HSPB1 | 3315 | -4.213 | 0.423 |
| YWHAB | 7529 | -3.443 | 0.423 |
| IGFBP1 | 3484 | 2.723 | 0.423 |
| PDE8A | 5151 | 2.848 | 0.423 |
| MYL9 | 10398 | -3.011 | 0.423 |
| 107987423 | 107987423 | 3.446 | 0.423 |
| OIT3 | 170392 | -3.170 | 0.423 |
| CLC | 1178 | 4.309 | 0.423 |
| CTSZ | 1522 | -3.224 | 0.423 |
| HSPA1L | 3305 | -3.115 | 0.423 |
| VASP | 7408 | -3.301 | 0.423 |
| ACTBL2 | 345651 | -5.677 | 0.423 |
| UTY | 7404 | -3.318 | 0.423 |
| GMFG | 9535 | -3.550 | 0.423 |
| GHR | 2690 | -2.937 | 0.423 |
| LAMA2 | 3908 | -1.647 | 0.423 |
| ENDOD1 | 23052 | -2.573 | 0.423 |
| UNC5B | 219699 | -3.793 | 0.423 |
| SPTA1 | 6708 | -2.810 | 0.423 |
| EIF5A | 1984 | -2.765 | 0.423 |
| ERBB2 | 2064 | 3.997 | 0.423 |
| SDPR | 8436 | -2.639 | 0.423 |
| GALNT2 | 2590 | -2.231 | 0.423 |
| TUBB1 | 81027 | -3.808 | 0.423 |
| LILRB1 | 10859 | -2.612 | 0.423 |
| TUBA1B | 10376 | -2.605 | 0.423 |
| CR1 | 1378 | -2.437 | 0.423 |
| PRAP1 | 118471 | -3.651 | 0.423 |
| GGT2 | 728441 | 1.958 | 0.423 |
| OSCAR | 126014 | 2.669 | 0.423 |
| CADM1 | 23705 | 3.262 | 0.423 |
| ANXA2 | 302 | -3.584 | 0.423 |
| HSP90AB1 | 3326 | -2.868 | 0.423 |
| CECR1 | 51816 | 3.510 | 0.423 |
| PIP | 5304 | -5.069 | 0.423 |
| IMPAD1 | 54928 | -2.234 | 0.423 |
| SOD1 | 6647 | 2.812 | 0.423 |
| UGT8 | 7368 | -4.537 | 0.423 |
| CBLN4 | 140689 | 4.054 | 0.423 |
| MYO1F | 4542 | -5.218 | 0.423 |
| NME1 | 4830 | -3.914 | 0.423 |
| CA6 | 765 | 3.309 | 0.423 |
| ASXL3 | 80816 | 3.720 | 0.423 |
| CALML3 | 810 | -4.624 | 0.423 |
| DSP | 1832 | -3.408 | 0.423 |
| FGFR4 | 2264 | -1.258 | 0.423 |
| GGT1 | 2678 | 1.718 | 0.423 |
| HLA-E | 3133 | -3.070 | 0.423 |
| HSPA2 | 3306 | -3.087 | 0.423 |
| MYO1E | 4643 | -5.300 | 0.423 |
| PSMA7 | 5688 | 3.589 | 0.423 |
| RDX | 5962 | -2.956 | 0.423 |
| LOC102723996 | 102723996 | -3.692 | 0.423 |
| LRPPRC | 10128 | 2.540 | 0.423 |
| MYL12B | 103910 | -2.923 | 0.423 |
| NUP188 | 23511 | -2.795 | 0.423 |
| GSR | 2936 | 3.126 | 0.423 |
| HBD | 3045 | -4.428 | 0.423 |
| IGF1 | 3479 | 3.141 | 0.423 |
| IHH | 3549 | -3.520 | 0.423 |
| RARRES1 | 5918 | 4.037 | 0.423 |
| SAA4 | 6291 | 6.677 | 0.423 |
| TPM1 | 7168 | -4.672 | 0.423 |
| BPIFB1 | 92747 | 3.316 | 0.423 |
| LGALS7 | 3963 | -2.395 | 0.423 |
| LGALS7B | 653499 | -2.395 | 0.423 |
| MAN2A1 | 4124 | 2.317 | 0.423 |
| KRT72 | 140807 | -4.165 | 0.423 |
| EGFR | 1956 | -2.296 | 0.423 |
| ADA | 100 | 4.235 | 0.423 |
| FGL1 | 2267 | -2.889 | 0.423 |
| NUP214 | 8021 | 3.029 | 0.423 |
| AK1 | 203 | -3.483 | 0.423 |
| AOC2 | 314 | 2.898 | 0.423 |
| PTPRM | 5797 | -2.815 | 0.423 |
| TTC30B | 150737 | -3.464 | 0.423 |
| PARK7 | 11315 | -3.755 | 0.423 |
| S100A12 | 6283 | -3.267 | 0.423 |
| DSC1 | 1823 | -3.721 | 0.423 |
| ICOSLG | 23308 | -3.776 | 0.423 |
| JUP | 3728 | -2.160 | 0.423 |
| KDM6A | 7403 | -3.320 | 0.423 |
| ABCC10 | 89845 | -3.208 | 0.423 |
| COL5A2 | 1290 | -2.330 | 0.423 |
| FAM3C | 10447 | 3.527 | 0.423 |
| SMPDL3A | 10924 | -2.892 | 0.423 |
| MYL12A | 10627 | -2.873 | 0.423 |
| SVEP1 | 79987 | -1.773 | 0.423 |
| MCOLN3 | 55283 | -3.369 | 0.423 |
| CENPE | 1062 | -2.503 | 0.423 |
| PDGFRB | 5159 | -2.505 | 0.423 |
| FAM234A | 83986 | -2.376 | 0.423 |
| LOC102724197 | 102724197 | 1.871 | 0.423 |
| COL6A1 | 1291 | -0.290 | 0.423 |
| CTSB | 1508 | 0.239 | 0.423 |
| B3GNT8 | 374907 | 0.467 | 0.423 |
| GLIPR2 | 152007 | 0.947 | 0.423 |
| TIMP1 | 7076 | -1.005 | 0.423 |
| C1QTNF3 | 114899 | -0.347 | 0.423 |
| PTPRK | 5796 | 0.152 | 0.423 |
| TXNDC15 | 79770 | -0.297 | 0.423 |
| SSC5D | 284297 | -0.191 | 0.423 |
| FCGR2C | 9103 | 0.233 | 0.423 |
| MARCO | 8685 | -0.077 | 0.423 |
| FCGR2A | 2212 | 0.233 | 0.423 |
| HTRA1 | 5654 | 0.031 | 0.423 |
| CPQ | 10404 | -0.091 | 0.423 |
| NCAM2 | 4685 | -0.025 | 0.423 |
| MB | 4151 | -3.391 | 0.422 |
| ATF6 | 22926 | 2.989 | 0.421 |
| APOC1 | 341 | 0.301 | 0.419 |
| FTL | 2512 | -3.997 | 0.418 |
| KIT | 3815 | -3.103 | 0.418 |
| ANPEP | 290 | 0.275 | 0.416 |
| FCGR3B | 2215 | 4.523 | 0.415 |
| COLEC10 | 10584 | 3.985 | 0.414 |
| ORM2 | 5005 | -0.317 | 0.412 |
| COL18A1 | 80781 | -2.635 | 0.410 |
| CTSG | 1511 | -3.469 | 0.410 |
| KRT1 | 3848 | -0.677 | 0.410 |
| LAMB1 | 3912 | -3.289 | 0.408 |
| C2 | 717 | -0.115 | 0.408 |
| IGFALS | 3483 | -0.156 | 0.408 |
| MMRN2 | 79812 | -3.804 | 0.408 |
| HSPA5 | 3309 | -0.599 | 0.407 |
| APP | 351 | -4.473 | 0.407 |
| DKK3 | 27122 | -3.607 | 0.407 |
| CR2 | 1380 | -2.516 | 0.405 |
| PHF21A | 51317 | 5.001 | 0.403 |
| FAH | 2184 | -3.160 | 0.403 |
| C1QB | 713 | 0.181 | 0.401 |
| CFHR5 | 81494 | -5.028 | 0.398 |
| SERPINA1 | 5265 | 0.285 | 0.398 |
| BCHE | 590 | -0.333 | 0.395 |
| F10 | 2159 | -0.122 | 0.393 |
| ERP44 | 23071 | -3.183 | 0.392 |
| RTN4RL2 | 349667 | -3.715 | 0.391 |
| EPHA4 | 2043 | -2.547 | 0.390 |
| GGH | 8836 | -0.124 | 0.388 |
| CD14 | 929 | -0.269 | 0.388 |
| AOC3 | 8639 | 3.022 | 0.388 |
| SEPP1 | 6414 | -0.021 | 0.386 |
| TGFBI | 7045 | -0.851 | 0.386 |
| SPARCL1 | 8404 | -0.440 | 0.385 |
| PAM | 5066 | -3.155 | 0.381 |
| C1QC | 714 | 0.080 | 0.377 |
| KRT14 | 3861 | -1.579 | 0.375 |
| CFB | 629 | 0.133 | 0.375 |
| PROCR | 10544 | -0.971 | 0.374 |
| SELENBP1 | 8991 | -4.753 | 0.369 |
| GOLM1 | 51280 | -3.260 | 0.369 |
| APOD | 347 | 0.402 | 0.369 |
| C1RL | 51279 | -0.497 | 0.368 |
| MRC2 | 9902 | -2.512 | 0.368 |
| ITGB1 | 3688 | -2.936 | 0.364 |
| ROBO4 | 54538 | 2.433 | 0.364 |
| SERPINA6 | 866 | -0.190 | 0.364 |
| MAN1A1 | 4121 | -0.069 | 0.363 |
| ORM1 | 5004 | -0.124 | 0.362 |
| CRTAC1 | 55118 | -0.383 | 0.350 |
| PEPD | 5184 | -1.391 | 0.349 |
| ITIH2 | 3698 | 0.123 | 0.347 |
| FLT4 | 2324 | -3.333 | 0.347 |
| HRNR | 388697 | -1.281 | 0.345 |
| F11 | 2160 | 0.220 | 0.345 |
| C7 | 730 | -0.093 | 0.345 |
| VWF | 7450 | 0.554 | 0.345 |
| SAA2 | 6289 | 3.483 | 0.344 |
| F13A1 | 2162 | 0.637 | 0.342 |
| PRKCSH | 5589 | -4.080 | 0.341 |
| CHL1 | 10752 | -0.431 | 0.338 |
| ERAP2 | 64167 | -2.528 | 0.338 |
| TPM4 | 7171 | -5.675 | 0.336 |
| F12 | 2161 | -0.256 | 0.333 |
| PTPRF | 5792 | -3.677 | 0.332 |
| LRP1 | 4035 | -2.163 | 0.329 |
| PROZ | 8858 | -0.527 | 0.328 |
| CRP | 1401 | 0.427 | 0.325 |
| VCL | 7414 | -5.056 | 0.321 |
| ADGRG6 | 57211 | 3.035 | 0.319 |
| CD44 | 960 | -0.183 | 0.319 |
| APOA2 | 336 | 0.722 | 0.317 |
| AMBP | 259 | -0.267 | 0.314 |
| APOH | 350 | 0.381 | 0.313 |
| COLEC11 | 78989 | 0.514 | 0.310 |
| SERPING1 | 710 | -0.124 | 0.309 |
| IGFBP3 | 3486 | -0.314 | 0.309 |
| PROC | 5624 | -0.250 | 0.301 |
| CALR | 811 | -5.328 | 0.298 |
| LYVE1 | 10894 | -0.649 | 0.298 |
| S100A6 | 6277 | -5.771 | 0.294 |
| LAMP2 | 3920 | -0.288 | 0.294 |
| CFD | 1675 | -0.159 | 0.293 |
| SLC3A2 | 6520 | -0.492 | 0.290 |
| CACNA2D1 | 781 | -1.671 | 0.287 |
| CHGA | 1113 | -5.803 | 0.285 |
| PCOLCE | 5118 | -1.038 | 0.282 |
| S100A4 | 6275 | -5.510 | 0.281 |
| HSPA8 | 3312 | -7.349 | 0.278 |
| CD5L | 922 | 5.627 | 0.275 |
| KRT5 | 3852 | -2.274 | 0.274 |
| RPS27A | 6233 | -5.290 | 0.273 |
| S100A7A | 338324 | -4.113 | 0.272 |
| S100A7 | 6278 | -4.113 | 0.272 |
| UBA52 | 7311 | -5.216 | 0.271 |
| PRDX2 | 7001 | -1.757 | 0.268 |
| AFM | 173 | -0.168 | 0.268 |
| S100A9 | 6280 | -1.355 | 0.265 |
| CST3 | 1471 | -0.181 | 0.262 |
| GP5 | 2814 | -7.219 | 0.260 |
| UBB | 7314 | -4.882 | 0.259 |
| C1QA | 712 | 1.049 | 0.257 |
| FLG2 | 388698 | -4.530 | 0.257 |
| PVR | 5817 | -0.546 | 0.256 |
| YWHAZ | 7534 | -2.618 | 0.255 |
| LRG1 | 116844 | -0.092 | 0.255 |
| SERPINA10 | 51156 | -0.346 | 0.255 |
| CD163 | 9332 | 0.857 | 0.252 |
| CDH13 | 1012 | -0.776 | 0.250 |
| GC | 2638 | -0.239 | 0.247 |
| GNPTG | 84572 | 0.936 | 0.247 |
| F5 | 2153 | 0.300 | 0.244 |
| FUCA2 | 2519 | -5.031 | 0.243 |
| ICAM2 | 3384 | -1.156 | 0.241 |
| BST1 | 683 | -5.516 | 0.240 |
| DCD | 117159 | -0.916 | 0.236 |
| UBC | 7316 | -4.332 | 0.236 |
| NRP1 | 8829 | -0.424 | 0.235 |
| AGT | 183 | -0.395 | 0.234 |
| ABI3BP | 25890 | -0.737 | 0.229 |
| HSPA6 | 3310 | 6.737 | 0.227 |
| CNTFR | 1271 | -5.978 | 0.227 |
| DSG2 | 1829 | -1.230 | 0.227 |
| IL1RAP | 3556 | -1.379 | 0.225 |
| KRT9 | 3857 | -1.073 | 0.224 |
| GSTO1 | 9446 | -8.078 | 0.224 |
| C4BPB | 725 | 1.137 | 0.222 |
| DBH | 1621 | -0.206 | 0.219 |
| MSN | 4478 | 5.066 | 0.219 |
| DSG1 | 1828 | -6.499 | 0.218 |
| PTPRJ | 5795 | -0.996 | 0.216 |
| S100A8 | 6279 | -0.727 | 0.215 |
| MMP2 | 4313 | -0.488 | 0.212 |
| DPEP2 | 64174 | -0.662 | 0.211 |
| ITGA2B | 3674 | -6.878 | 0.209 |
| FETUB | 26998 | -0.539 | 0.206 |
| ACTA1 | 58 | -1.743 | 0.204 |
| ACTC1 | 70 | -1.743 | 0.204 |
| PPIA | 5478 | -2.540 | 0.202 |
| ACTN1 | 87 | -6.495 | 0.201 |
| CALML5 | 51806 | -9.796 | 0.201 |
| CAT | 847 | -2.150 | 0.201 |
| KRT4 | 3851 | 8.597 | 0.200 |
| BTD | 686 | -0.398 | 0.199 |
| FCGBP | 8857 | 0.472 | 0.198 |
| MYL6 | 4637 | -6.634 | 0.198 |
| ZFP14 | 57677 | 6.754 | 0.194 |
| ITGB3 | 3690 | -6.657 | 0.194 |
| CTSF | 8722 | -6.312 | 0.193 |
| MINPP1 | 9562 | -0.440 | 0.193 |
| CDH1 | 999 | -5.747 | 0.193 |
| CFHR4 | 10877 | -9.780 | 0.192 |
| APOA4 | 337 | -0.318 | 0.192 |
| FLG | 2312 | -5.243 | 0.192 |
| MMRN1 | 22915 | -1.361 | 0.192 |
| SERPINE1 | 5054 | -6.076 | 0.191 |
| CPN1 | 1369 | -0.060 | 0.191 |
| NEBL | 10529 | 8.483 | 0.190 |
| CTSH | 1512 | -6.804 | 0.189 |
| CAP1 | 10487 | -7.617 | 0.189 |
| HLA-A | 3105 | -0.819 | 0.189 |
| PPIB | 5479 | -7.303 | 0.189 |
| SRGN | 5552 | -11.116 | 0.189 |
| TUBA4A | 7277 | -5.995 | 0.189 |
| PRDX1 | 5052 | -6.308 | 0.189 |
| RARRES2 | 5919 | 7.987 | 0.188 |
| FBN1 | 2200 | 3.708 | 0.188 |
| KRT3 | 3850 | -7.285 | 0.188 |
| SERPINA4 | 5267 | -0.271 | 0.187 |
| KRT6A | 3853 | -10.455 | 0.187 |
| PKM | 5315 | -7.061 | 0.187 |
| SPTB | 6710 | -4.851 | 0.187 |
| ALAD | 210 | -7.836 | 0.187 |
| AIM1L | 55057 | 8.667 | 0.186 |
| CSF1R | 1436 | -0.809 | 0.186 |
| ARHGDIA | 396 | -7.910 | 0.185 |
| TMEM189-UBE2V1 | 387522 | -5.559 | 0.185 |
| UBE2V2 | 7336 | -6.255 | 0.185 |
| JCHAIN | 3512 | 9.416 | 0.185 |
| UBE2V1 | 7335 | -6.473 | 0.185 |
| CALM2 | 805 | -7.659 | 0.185 |
| CALM1 | 801 | -7.692 | 0.185 |
| TPM3 | 7170 | -8.961 | 0.185 |
| CALM3 | 808 | -7.782 | 0.185 |
| SELP | 6403 | -6.879 | 0.185 |
| SLC4A1 | 6521 | -7.266 | 0.184 |
| PNP | 4860 | -8.177 | 0.184 |
| PZP | 5858 | -0.269 | 0.184 |
| WDR1 | 9948 | -6.428 | 0.184 |
| PMEL | 6490 | -6.109 | 0.184 |
| HSD11B1L | 374875 | -7.934 | 0.184 |
| PCSK1N | 27344 | 7.493 | 0.184 |
| TLN1 | 7094 | -8.345 | 0.184 |
| SFTPA1 | 653509 | -6.475 | 0.184 |
| SFTPA2 | 729238 | -6.475 | 0.184 |
| HPRT1 | 3251 | -6.283 | 0.184 |
| TUBB4A | 10382 | -4.884 | 0.184 |
| TUBB4B | 10383 | -4.884 | 0.184 |
| TUBB2B | 347733 | -4.884 | 0.184 |
| TUBB2A | 7280 | -4.952 | 0.184 |
| TUBB | 203068 | -5.015 | 0.184 |
| MTPN | 136319 | -7.061 | 0.184 |
| SH3BGRL3 | 83442 | -7.521 | 0.184 |
| CFHR3 | 10878 | 13.352 | 0.184 |
| YWHAH | 7533 | -7.606 | 0.184 |
| ADAMDEC1 | 27299 | -6.796 | 0.184 |
| MYH9 | 4627 | -6.398 | 0.184 |
| LOC100653049 | 100653049 | -5.440 | 0.184 |
| KRT34 | 3885 | -5.440 | 0.184 |
| KRT33A | 3883 | -5.538 | 0.184 |
| HYAL1 | 3373 | 6.858 | 0.184 |
| PRDX6 | 9588 | -7.228 | 0.184 |
| PSMA5 | 5686 | -6.476 | 0.184 |
| GGCT | 79017 | -7.077 | 0.184 |
| FERMT3 | 83706 | -7.694 | 0.184 |
| PARVB | 29780 | -6.562 | 0.184 |
| BLMH | 642 | 9.682 | 0.184 |
| FAM50A | 9130 | -9.400 | 0.184 |
| MANBA | 4126 | -4.822 | 0.184 |
| LDHB | 3945 | -0.948 | 0.182 |
| KLKB1 | 3818 | 0.246 | 0.181 |
| CLSTN1 | 22883 | -6.127 | 0.180 |
| MMP9 | 4318 | -6.703 | 0.179 |
| KRT8 | 3856 | 7.935 | 0.177 |
| TXN | 7295 | -9.224 | 0.177 |
| HBA1 | 3039 | -1.711 | 0.177 |
| HBA2 | 3040 | -1.711 | 0.177 |
| TGOLN2 | 10618 | -0.504 | 0.175 |
| APOF | 319 | -0.280 | 0.174 |
| TIMP2 | 7077 | -0.675 | 0.172 |
| TGFB1 | 7040 | -7.864 | 0.170 |
| THBS1 | 7057 | -2.636 | 0.165 |
| TPI1 | 7167 | -8.880 | 0.162 |
| C8A | 731 | -0.388 | 0.155 |
| TMSB4X | 7114 | -8.255 | 0.154 |
| PF4 | 5196 | -1.366 | 0.148 |
| PF4V1 | 5197 | -1.366 | 0.148 |
| ARHGAP35 | 2909 | -1.707 | 0.147 |
| PGK1 | 5230 | -5.576 | 0.145 |
| PPBP | 5473 | -1.852 | 0.144 |
| F7 | 2155 | -0.320 | 0.140 |
| ALDOA | 226 | -1.786 | 0.139 |
| ATRN | 8455 | -0.033 | 0.128 |
| ACTB | 60 | -2.087 | 0.126 |
| ACTG1 | 71 | -2.087 | 0.126 |
| GAPDH | 2597 | -9.162 | 0.122 |
| SERPINA5 | 5104 | -0.316 | 0.118 |
| CDH5 | 1003 | -0.457 | 0.111 |
| HBB | 3043 | -1.837 | 0.111 |
| TAGLN2 | 8407 | -8.600 | 0.110 |
| CALU | 813 | -10.260 | 0.104 |
| CA2 | 760 | -2.030 | 0.104 |
| BLVRB | 645 | -2.393 | 0.103 |
| GPX3 | 2878 | -0.588 | 0.101 |
| DMKN | 93099 | -1.297 | 0.099 |
| LBP | 3929 | -0.416 | 0.095 |
| CLEC3B | 7123 | -0.303 | 0.095 |
| LTBP1 | 4052 | -7.225 | 0.082 |
| ECM1 | 1893 | 1.531 | 0.080 |
| FN1 | 2335 | 0.350 | 0.077 |
| PLXDC2 | 84898 | -0.290 | 0.072 |
| CA1 | 759 | -1.735 | 0.072 |
| APOB | 338 | 0.257 | 0.069 |
| C8G | 733 | 0.229 | 0.063 |
| A1BG | 1 | 0.083 | 0.063 |
| TNXB | 7148 | -0.617 | 0.062 |
| TNC | 3371 | -1.015 | 0.061 |
| SBSN | 374897 | -0.999 | 0.058 |
| FCN2 | 2220 | -0.256 | 0.057 |
| CPB2 | 1361 | -0.304 | 0.054 |
| SPARC | 6678 | -1.859 | 0.054 |
| RNASE4 | 6038 | -0.593 | 0.052 |
| ITIH3 | 3699 | -0.153 | 0.051 |
| MCAM | 4162 | -0.491 | 0.048 |
| SELL | 6402 | -0.433 | 0.038 |
| APOA1 | 335 | 0.095 | 0.037 |
| PRG4 | 10216 | 0.212 | 0.037 |
| MASP2 | 10747 | 0.288 | 0.018 |
| CPN2 | 1370 | -0.266 | 0.012 |
| APOE | 348 | 0.479 | 0.006 |
